# TRIAD3/RNF216 mutations associated with Gordon Holmes syndrome lead to synaptic and cognitive impairments via Arc misregulation

**DOI:** 10.1111/acel.12551

**Published:** 2016-12-20

**Authors:** Nilofer Husain, Qiang Yuan, Yi‐Chun Yen, Olga Pletnikova, Dong Qianying Sally, Paul Worley, Zoë Bichler, H. Shawn Je

**Affiliations:** ^1^ Signature Program in Neuroscience and Behavioral DisordersDuke‐NUS Medical School Singapore8 College RoadSingapore169857Singapore; ^2^ Department of PathologyJohns Hopkins University School of MedicineBaltimoreMD21205USA; ^3^ Behavioral Neuroscience LaboratoryNational Neuroscience Institute11 Jalan Tan Tock Seng308433SingaporeSingapore; ^4^ Solomon H. Snyder Department of NeuroscienceJohns Hopkins University School of MedicineBaltimoreMD21205USA; ^5^ Department of PhysiologyYong Loo Lin School of MedicineNational University of SingaporeSingapore117597Singapore

**Keywords:** activity‐regulated cytoskeletal protein (Arc/Arg 3.1), behavior, dementia, Gordon Holmes syndrome, learning and memory, synapse, TRIAD3, ubiquitination

## Abstract

Multiple loss‐of‐function mutations in *TRIAD3* (a.k.a. RNF216) have recently been identified in patients suffering from Gordon Holmes syndrome (GHS), characterized by cognitive decline, dementia, and movement disorders. TRIAD3A is an E3 ubiquitin ligase that recognizes and facilitates the ubiquitination of its target for degradation by the ubiquitin‐proteasome system (UPS). Here, we demonstrate that two of these missense substitutions in TRIAD3 (R660C and R694C) could not regulate the degradation of their neuronal target, activity‐regulated cytoskeletal‐associated protein (Arc/Arg 3.1), whose expression is critical for synaptic plasticity and memory. The synaptic deficits due to the loss of endogenous TRIAD3A could not be rescued by TRIAD3A harboring GHS‐associated missense mutations. Moreover, we demonstrate that the loss of endogenous TRIAD3A in the mouse hippocampal CA1 region led to deficits in spatial learning and memory. Finally, we show that these missense mutations abolished the interaction of TRIAD3A with Arc, disrupting Arc ubiquitination, and consequently Arc degradation. Our current findings of Arc misregulation by TRIAD3A variants suggest that loss‐of‐function mutations in TRIAD3A may contribute to dementia observed in patients with GHS driven by dysfunctional UPS components, leading to cognitive impairments through the synaptic protein Arc.

## Introduction

Dementia encompasses diverse diseases that share cognitive deficits in areas involving executive function, attention, memory, and recognition (Margolin *et al*., 2013). Previous studies have shown that protein degradation by the ubiquitin‐proteasome system (UPS) is critical for the formation of long‐term memory in inhibitory avoidance test and memory reorganization after fear memory retrieval (Lopez‐Salon *et al*., 2001; Lee *et al*., 2008). Although UPS malfunction has been reported in Alzheimer's disease (AD), the most common form of dementia (Pasqualetti *et al*., 2015), the exact molecular identity that links UPS with dementia is still elusive.

The most common form of dementia is AD; however, other forms exist, including frontotemporal dementia, Lewy body dementia, and vascular dementia (Pasqualetti *et al*., 2015). Although rare, familial forms of dementia that are caused by heritable mutation(s) in certain genes provide valuable insights into the underlying cellular mechanisms of dementia. Gordon Holmes syndrome (GHS) is an adult‐onset disorder characterized by cognitive decline, dementia, and other clinical features such as ataxia and hypogonadotropism (Margolin *et al*., 2013). GHS was first described in 1907 by Holmes in a family of one sister and three brothers with symptoms of ataxia and hypogonadism (Holmes, 1907). Patients with GHS have consanguineous parents in most of the cases, indicating an autosomal recessive mode of inheritance (Berciano *et al*., 1982; Abs *et al*., 1990; Seminara *et al*., 2002; Margolin *et al*., 2013; Alqwaifly & Bohlega, 2016). Intriguingly, multiple novel mutations (missense and nonsense mutations) in *TRIAD3* (*RNF216*), a gene encoding E3 ubiquitin ligase that recognizes the target protein and conjugates ubiquitin for target protein degradation, were identified in patients suffering from GHS (Margolin *et al*., 2013). Coincidentally, it was recently found that the function of the TRIAD3A protein in neurons is to regulate synaptic transmission and plasticity by acting as an E3 ubiquitin ligase of activity‐regulated cytoskeletal protein (Arc/Arg 3.1) (Mabb *et al*., 2014). Arc is an immediate early gene product that is involved in multiple forms of synaptic plasticity, such as long‐term potentiation, long‐term depression (LTD), and homeostatic scaling, all of which are implicated in normal cognitive function, including learning and memory. The roles of Arc in LTD and homeostatic scaling have been attributed to its ability to enhance endocytosis of synaptic AMPA‐type glutamate receptors (Shepherd *et al*., 2006; Waung *et al*., 2008). Therefore, as TRIAD3A maintains the appropriate level of Arc required for synaptic transmission and plasticity, its dysfunction potentially underlies the cognitive deficits observed in patients with dementia (Wu *et al*., 2011).

Here, we identified that missense substitutions in TRIAD3 (both R660C and R694C) resulted in defective Arc ubiquitination and degradation. As a result, the decreased synaptic strength due to TRIAD3A knockdown in neurons could not be rescued by the TRIAD3A missense variants, thereby causing aberrant synaptic transmission. Furthermore, *in vivo* knockdown of endogenous TRIAD3A in the CA1 region of the mouse hippocampus simulating the loss‐of‐function dementia‐related *TRIAD3A* mutations led to deficits in spatial learning and memory. Taken together, our results demonstrate that the loss‐of‐function dementia‐related mutations in *TRIAD3A* or reduced endogenous TRIAD3A protein levels may contribute to cognitive deficits in dementia through misregulation of Arc degradation in neurons.

## Results

### TRIAD3/RNF216 missense variants found in patients with GHS failed to degrade the Arc protein

**Figure 1:**
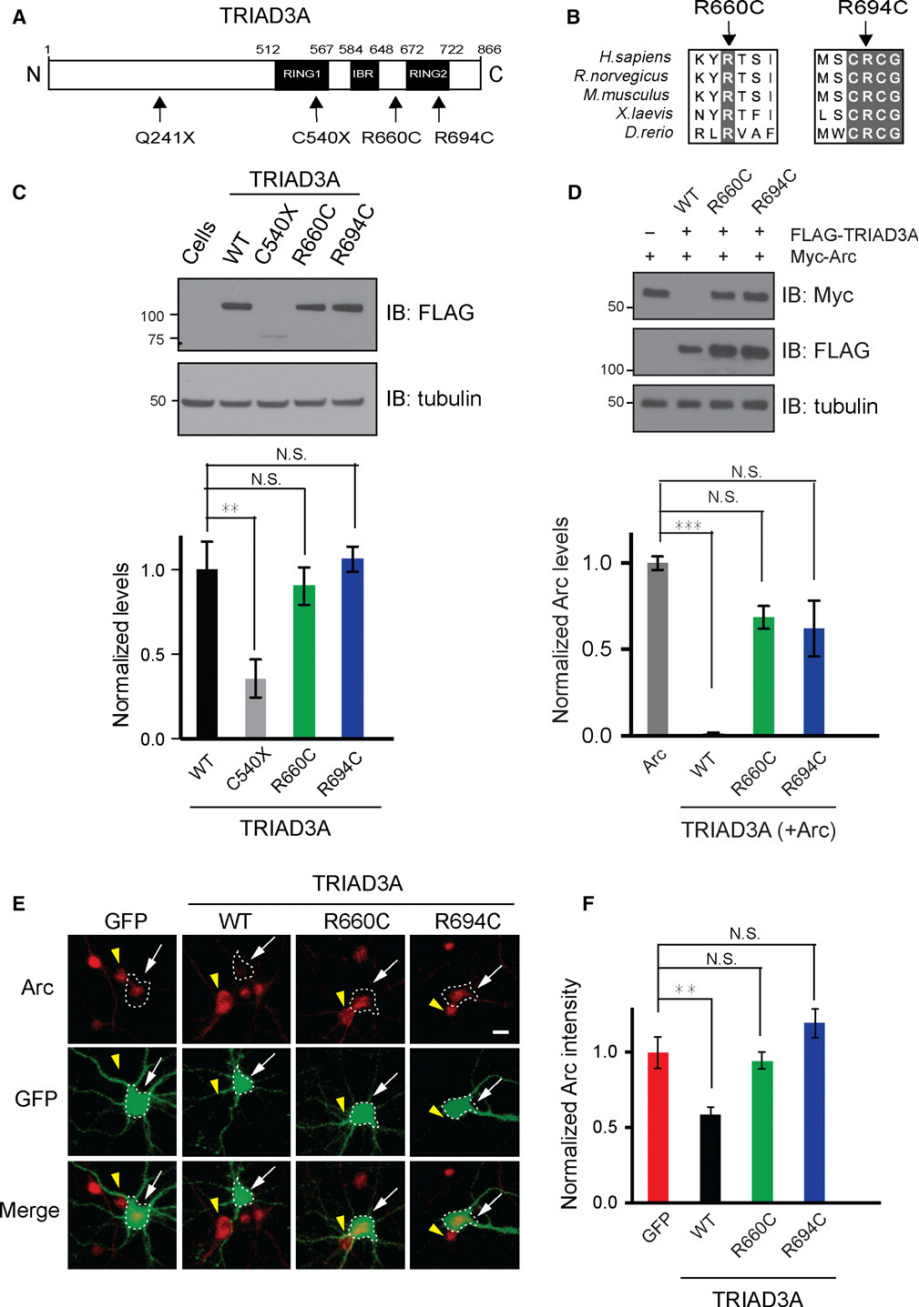
TRIAD3A variants do not degrade Arc. (A) TRIAD3A is an 866‐aa‐long protein (white) consisting of two RING domains, RING1 and RING2, which are separated by an in‐between RING fingers (IBR) domain. The positions of the four variants identified in patients with GHS are labeled. (B) TRIAD3A residues R660 and R694 are conserved across different organisms (human, rat, mouse, frog, and zebrafish). (C) Top panel: Western blot analyses were performed on HEK293T lysates overexpressing FLAG‐tagged TRIAD3A variants and blotted with an anti‐FLAG antibody and an anti‐tubulin antibody (loading control). Bottom panel: Quantification of TRIAD3A and TRIAD3A variants are depicted as the mean ± SEM (one‐way ANOVA, **P < 0.01, *n* = 4). (D) Top panel: HEK293T cells were cotransfected with 0.25 μg of Arc and 1 μg of WT TRIAD3A or TRIAD3A variants as indicated. Reduction in Arc levels is observed when Arc (α‐Myc) is co‐expressed with WT TRIAD3A, but not TRIAD3A point mutants. Bottom panel: Quantification of Arc levels when 1.0 μg WT TRIAD3A or TRIAD3A variants were overexpressed in HEK293T cells. The data represent the mean ± SEM. Statistical significance was assessed by one‐way ANOVA and Student's *t*‐test (***P < 0.001), *n* = 3. (E) Representative images of cultured cortical neurons transfected with GFP, WT TRIAD3A, TRIAD3A‐R660C (R660C), or TRIAD3A‐R694C (R694C). The white arrows indicate a transfected neuron whereas the yellow arrowhead indicates an untransfected neuron. Scale bar, 5 μm. (F) Histograms of results for GFP (*n* = 19), TRIAD3A (*n* = 34), R660C (*n* = 48), or R694C (*n* = 32). All histogram data are shown as the mean ± SEM. Statistical significance was assessed by one‐way ANOVA (*P < 0.05, **P < 0.01, ***P < 0.001).

Recently, four mutations [two nonsense mutations (Q184X and C540X) and two missense mutations (R660C and R694C)] in the gene encoding *TRIAD3/RNF216* were identified in patients with GHS (Fig. 1A; Margolin *et al*., 2013). The two missense mutations (R660C and R694C) reside near (R660C) or within (R694C) the RING2 region of the C‐terminal RING1‐between‐RING2 (RBR) domain of TRIAD3. These residues (R660 and R694) are evolutionarily conserved across different species, including humans, rodents, zebrafishes, and frogs (Fig. 1B).

TRIAD3 is expressed as five isoforms (TRIAD3A, B, C, D, and E). We focused on TRIAD3A because it is the most abundantly expressed isoform in the brain (Chuang & Ulevitch, 2004; Mabb *et al*., 2014). We generated expression constructs for TRIAD3A wild‐type (WT) and variants with an N‐terminal FLAG tag. Expression of these constructs in human embryonic kidney 293T (HEK293T) cells followed by Western blot analysis with a specific antibody against FLAG revealed that the TRIAD3A WT and mutant proteins had identical molecular weights and expression levels (WT vs. R660C, *P* = 0.9252; WT vs. R694C, *P* = 0.9783; *n* = 4; Fig. 1C). In contrast, the TRIAD3A nonsense variant (C540X) showed a significant (~65%) reduction in protein expression (WT vs. C540X, *P* = 0.0078; Fig. 1C).

To test whether either of the two missense variants of TRIAD3A (R660C and R694C) mediate Arc protein degradation, HEK293T cells were cotransfected with a FLAG‐tagged expression construct of either the WT TRIAD3A or a variant (R660C or R694C) together with a Myc‐tagged Arc expression construct and subjected to Western blot analyses. Previously, increasing amounts of TRIAD3A were shown to decrease Arc protein levels progressively through TRIAD3A‐mediated Arc ubiquitination and subsequent degradation by the UPS (Mabb *et al*., 2014). Intriguingly, we found that both the R660C and R694C variants failed to decrease Arc protein levels compared with TRIAD3A WT (>99%) (WT + Arc vs. Arc, *P* = 0.0002; R660C + Arc vs. Arc, *P* = 0.110; and R694C + Arc vs. Arc, *P* = 0.051; *n* = 4; Fig. 1D).

Given that TRIAD3A WT overexpression reduces endogenous Arc protein levels in cultured neurons (Mabb *et al*., 2014), cortical neurons were transfected with either WT or R660C and R694C TRIAD3A variants with GFP (Green Fluorescent Protein) cDNA and were immunostained with a specific antibody against Arc (Fig. 1E). Quantification of Arc immunoreactivity revealed a 41% reduction in endogenous Arc levels when neurons overexpressed TRIAD3A WT (*P* = 0.0034; Fig. 1F). However, neither R660C nor R694C TRIAD3A reduced endogenous Arc protein levels (GFP vs. R660C, *P* = 0.96; GFP vs. R694C, *P* = 0.31; *n* = 19–49; Fig. 1E,F). These results indicate that missense variants of TRIAD3A (R660C and R694C) linked to dementia are loss‐of‐function mutants in their ability to regulate Arc protein degradation.

### Both R660C and R694C TRIAD3A variants failed to increase basal synaptic transmission through AMPA receptor regulation

**Figure 2:**
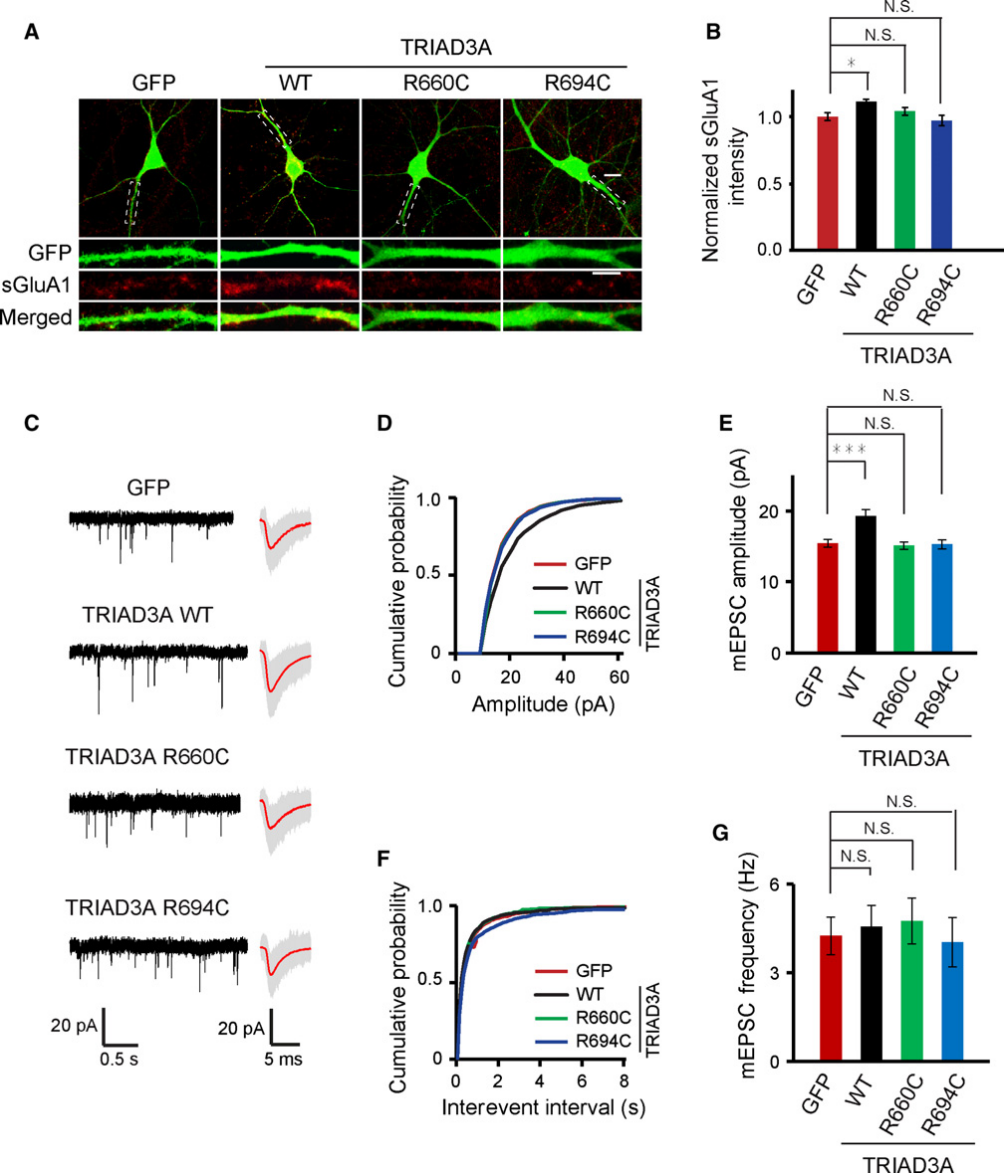
Increase in synaptic strength is impaired by the overexpression of the TRIAD3A variants. (A) Representative images of cortical neurons overexpressing GFP (control), wild‐type TRIAD3A (WT), TRIAD3A‐R660C (R660C), or TRIAD3A‐R694C (R694C) obtained after live antibody feeding with an anti‐GluA1 antibody are shown. Scale bars: 10 μm in the top panel and 5 μm in the bottom panel, respectively. (B) Quantification of surface GluA1 levels as the mean ± SEM is shown (*P* < 0.05). (C) Representative gap‐free recorded traces (left) and averaged mEPSC waveform (right) are shown for GFP (control), wild‐type TRIAD3A (WT), TRIAD3A‐R660C (R660C), or TRIAD3A‐R694C (R694C). In the averaged mEPSC waveform panel, gray traces indicate the overlaid raw traces and the red line indicates the average. (D,F) Cumulative plots and histograms (E,G) of mEPSC amplitude and frequency for GFP (*n* = 32), TRIAD3A (*n* = 36), R660C (*n* = 30), and R694C (*n* = 30). All histogram data are shown as the mean ± SEM. Statistical significance was assessed by one‐way ANOVA (*P < 0.05; ***P < 0.001).

TRIAD3A overexpression in neurons reduces Arc protein expression, resulting in decreased AMPA receptor endocytosis and increased surface AMPA receptor levels at excitatory synapses. Consistent with this model, TRIAD3A WT overexpression in neurons caused increased surface AMPA levels as revealed by surface labeling of the AMPA receptor subunit GluA1 (WT vs. GFP, *P* = 0.021, *n* = 9–11; Fig. 2A,B). In contrast, overexpression of TRIAD3A R660C or R694C failed to increase surface AMPA receptor levels in comparison with GFP (GFP vs. R660C, *P* = 0.76; GFP vs. R694C, *P* = 0.99; Fig. 2A,B).

To examine whether the modest increase in surface AMPA receptor levels observed upon TRIAD3A overexpression correlated with functional changes in AMPA receptor‐mediated basal synaptic transmission, we measured miniature excitatory postsynaptic currents (mEPSCs) at a resting membrane potential of −70 mV using whole‐cell patch clamp recordings. TRIAD3A WT overexpression increased mEPSC amplitudes significantly compared with neurons expressing GFP alone, without affecting mEPSC frequencies (GFP, 15.41 ± 0.54 pA, 4.25 ± 0.63 Hz, *n* = 32; TRIAD3A WT, 19.24 ± 0.94 pA, 4.56 ± 0.72 Hz, *n* = 36, *P* < 0.001 (amplitude); Fig. 2C–G). In contrast, overexpression of TRIAD3A R660C or R694C did not increase mEPSC amplitudes compared with neurons expressing GFP alone (TRIAD3A R660C, 15.09 ± 0.53 pA, 4.75 ± 0.78 Hz, *n* = 30, *P* = 0.39 relative to GFP (amplitude); TRIAD3A R694C, 15.28 ± 0.63 pA, 4.03 ± 0.83 Hz, *n* = 30 *P* = 0.907 relative to GFP (amplitude); Fig. 2C–G).

**Figure 3:**
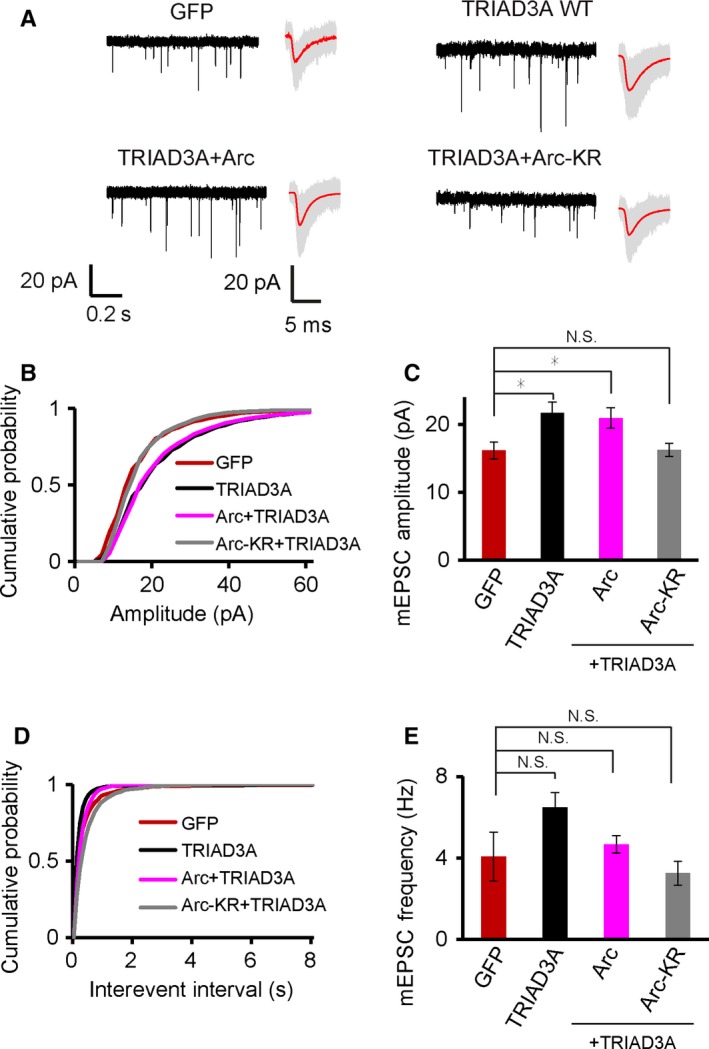
The increase in basal synaptic transmission by TRIAD3A overexpression is reversed by expression of Arc mutant that cannot be ubiquitinated. (A) Representative gap‐free recorded trace (left) and averaged mEPSC waveform (right) are shown for GFP, TRIAD3A‐WT (TRIAD3A) cotransfected with Arc WT (Arc) or Arc‐K268R/K269R (Arc‐KR). In the averaged mEPSC waveform panel, gray traces indicate the overlaid raw traces and the red line indicates the average. (B,D) Cumulative plot and (C,E) histograms of mEPSC amplitude and frequency for GFP (*n* = 14), TRIAD3A (*n* = 12), TRIAD3A + Arc (*n* = 16), and TRIAD3A + Arc‐KR (*n* = 15). All histogram data are shown as the mean ± SEM. Statistical significance was assessed by one‐way ANOVA (*P < 0.05).

Next, to test whether the increase in synaptic transmission due to TRIAD3A overexpression is mediated *via* Arc, we transfected cortical neurons with TRIAD3A, along with WT Arc and a ubiquitination‐defective Arc‐K268R/K269R (Arc‐KR) variant (Mabb *et al*., 2014). The overexpression of Arc‐KR restores Arc to the level of the control (GFP) as shown by Arc immunostaining (Fig. S1A,B). The overexpression of WT TRIAD3A or WT TRIAD3A with WT Arc (TRIAD3A + Arc) significantly increased mEPSC amplitudes compared with neurons expressing GFP, without affecting the frequencies (GFP, 16.15 ± 1.24 pA, 4.07 ± 1.19 Hz, *n* = 14; TRIAD3A, 21.72 ± 1.75 pA, 6.27 ± 0.75 Hz, *n* = 12; TRIAD3A + Arc, 21.38 ± 1.52 pA, 4.80 ± 0.43 Hz, *n* = 16; GFP vs. TRIAD3A, *P* = 0.04 (amplitude), *P* = 0.98 (frequency); GFP vs. TRIAD3A + Arc, *P* = 0.04 (amplitude), *P* = 0.99 (frequency); Fig. 3A–E). In contrast, overexpression of WT TRIAD3A with Arc‐KR prevented this increase in amplitude in comparison with the control GFP group (TRIAD3A + Arc‐KR, 16.47 ± 0.98 pA, 3.41 ± 0.61 Hz, *n* = 15; GFP vs. TRIAD3A + Arc‐KR, *P* = 1.00 (amplitude), *P* = 0.31 (frequency); Fig. 3A–E). These data indicate that the increased basal synaptic transmission induced by TRIAD3A overexpression was due to Arc ubiquitination and degradation in neurons.

### The decrease in synaptic strength induced by TRIAD3A knockdown could not be rescued by TRIAD3A R660C and R694C variants

Next, we investigated whether the R660C or R694C TRIAD3A variant could rescue the synaptic effects triggered by the loss of endogenous TRIAD3A in cortical neurons resulting from the expression of small hairpin RNA (shRNA) targeting TRIAD3A as shown previously using cultured hippocampal neurons (Mabb *et al*., 2014). The TRIAD3A shRNA (TRIAD3A‐sh) could efficiently knockdown exogenously expressed TRIAD3A in HEK293T cells, and this knockdown effect of TRIAD3A‐sh could be rescued by the co‐expression of an shRNA‐resistant TRIAD3A WT (R) expression construct (Fig. S2).

**Figure 4:**
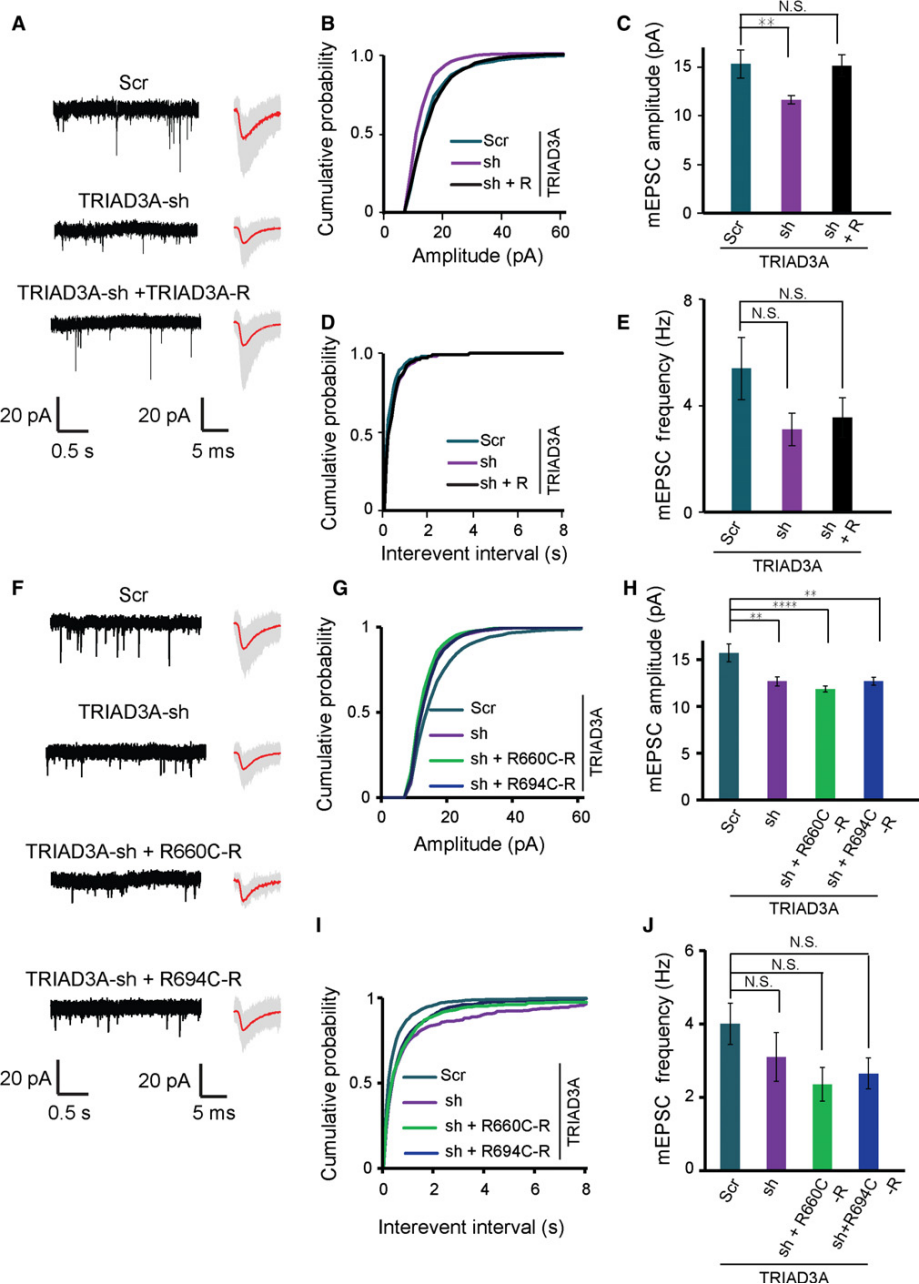
The decrease in synaptic strength by TRIAD3A knockdown cannot be rescued by TRIAD3A mutants. (A) Representative gap‐free recorded trace (left) and averaged mEPSC waveform (right) are shown for scrambled shRNA (Scr), TRIAD3A‐shRNA (TRIAD3A‐sh), or TRIAD3A‐shRNA cotransfected with shRNA‐resistant TRIAD3A (TRIAD3A‐sh + TRIAD3A‐R). In the averaged mEPSC waveform panel, gray traces indicate the overlaid raw traces and the red line indicates the average. (B,D) Cumulative plot and (C,E) histograms of mEPSC amplitude and frequency for Scr (*n* = 14), sh (*n* = 14), and sh + TRIAD3A‐R (*n* = 12; **P < 0.01). (F) Representative gap‐free recorded trace (left) and averaged mEPSC waveform (right) are shown for cortical neurons transfected with scrambled shRNA (Scr), TRIAD3A‐shRNA (TRIAD3A‐sh), or TRIAD3A‐shRNA cotransfected with shRNA‐resistant TRIAD3A variants (TRIAD3A‐sh + R660C‐R and TRIAD3A‐sh + R694C‐R). In the averaged mEPSC waveform panel, gray traces indicate the overlaid raw traces and the red line indicates the average. (G–J) mEPSC for knockdown rescue of the TRIAD3A variants (*n* = 25, 28, 21, 23 for Scr, sh, sh + R660C, and sh + R694C, respectively). All histogram data are shown as the mean ± SEM. Statistical significance was assessed by one‐way ANOVA (****P < 0.0001; **P < 0.01).

Previously, we showed that TRIAD3A knockdown in cultured hippocampal neurons resulted in Arc upregulation (Mabb *et al*., 2014). Endogenous TRIAD3A knockdown in neurons caused a significant decrease in mEPSC amplitudes but not frequencies (TRIAD3A‐sh, 11.64 ± 0.43 pA, 3.11 ± 0.61 Hz, *n* = 12; scrambled shRNA (Scr), 15.32 ± 1.44 pA, 5.39 ± 1.17 Hz, *n* = 14, *P* = 0.007 (amplitude) and *P* = 0.18 (frequency) relative to Scr; Fig. 4A–E). Importantly, the synaptic effects of TRIAD3A knockdown could be rescued by the co‐expression of TRIAD3A WT (R) (15.15 ± 1.13 pA, 3.56 ± 0.75 Hz, *n* = 12; *P* = 0.99 (amplitude) relative to Scr; Fig. 4A–E). Next, we tested whether an shRNA‐resistant R660C (R660C‐R) or R694C (R694C‐R) TRIAD3A variant could rescue TRIAD3A knockdown phenotypes of basal synaptic transmission. Neither the R660C nor R694C variant could rescue the TRIAD3A‐sh‐induced decrease in mEPSC amplitude (Scr, 15.71 ± 0.97 pA, 4.00 ± 0.56 Hz, *n* = 25; TRIAD3A‐sh, 12.68 ± 0.48 pA, 3.09 ± 0.67 Hz, *n* = 28, *P* = 0.001 (amplitude), *P* = 0.62 (frequency); TRIAD3A‐sh + R660C‐R, 11.85 ± 0.32 pA, 2.35 ± 0.46 Hz, *n* = 21, *P* < 0.0001 (amplitude), *P* = 0.18 (frequency); TRIAD3A‐sh + R694C‐R, 12.68 ± 0.42 pA, 2.65 ± 0.41 Hz; *P* = 0.002 (amplitude), *P* = 0.32 (frequency) relative to Scr; Fig. 4F–J). These results confirmed that the TRIAD3A R660C or R694C variants could not rescue TRIAD3A knockdown phenotypes of basal synaptic transmission.

To further test whether the decrease in synaptic transmission due to TRIAD3A knockdown was mediated *via* Arc, we transfected cortical neurons with TRIAD3A shRNA together with either Arc shRNA or scrambled shRNA. The knockdown of TRIAD3A alone (TRIAD3A sh + Scr) resulted in a 31% decrease in mEPSC amplitudes compared with the knockdown of both TRIAD3A and Arc (TRIAD3A sh + Arc sh) with no change in mEPSC frequencies (TRIAD3A sh + Scr, 14.39 ± 0.66 pA, 7.64 ± 1.38 Hz, TRIAD3A sh + Arc sh, 20.93 ± 1.45 pA, 10.19 ± 1.97 Hz, *n* = 20, *P* = 0.0002 (amplitude), *P* = 0.284 (frequency); Fig. S3). These data indicated that reduced basal synaptic transmission triggered by TRIAD3A knockdown was due to elevated Arc protein levels in neurons.

### TRIAD3A knockdown in the hippocampus impairs spatial memory

**Figure 5:**
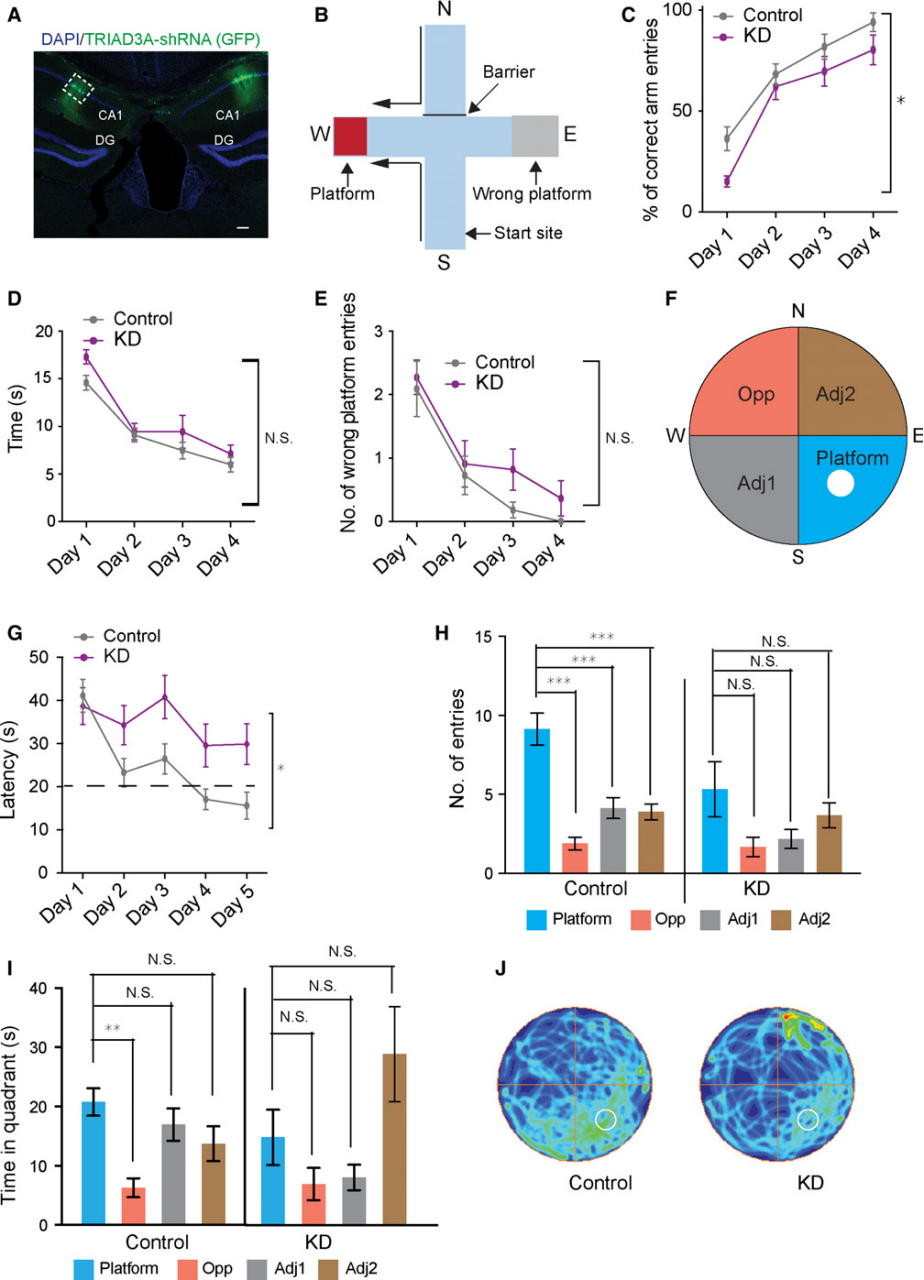
Loss of TRIAD3A in the CA1 region of the mouse hippocampus impairs spatial memory. (A) Coronal mouse brain section with the shRNA virus‐infected CA1 region as indicated by the GFP signal. Scale bar, 100 μm. (B) Schematic representation of the water‐cross maze experiment. The four arms are labeled north (N), south (S), east (E), and west (W). The location of the platform is shown. (C) The percentages of correct arm entries calculated from the six trials each day are shown. The KD group is deficient in locating the platform as accurately as the control group (two‐way ANOVA followed by Sidak's *post hoc* test, *P < 0.05). The time taken by the KD and control groups to reach the platform (latency) (D) and the number of wrong platform visits (E) over the 4 days of training are shown. Statistical significance was assessed by two‐way ANOVA and Student's *t*‐test (*n* = 11 for the KD group and *n* = 11 for the control group). (F) Scheme of the Morris water maze with the reference points used in the protocol and the names of the quadrants that are used for the analysis. The platform was located in the S‐E quadrant of the pool. (G) Latency to reach the platform within the 5 days of training is shown. The control group learned the location of the platform, but the KD group did not (two‐way ANOVA followed by Bonferroni *post hoc* comparison, *P < 0.05; control: *n* = 8; KD: *n* = 6). The dotted line indicates the threshold for the animal to acquire learning (20 s to reach the platform). Number of entries (H) and time spent (I) in the different quadrants of the pool during the probe test on the 6th day, 24 h after the last learning trial. Data are presented as the means ± SEM. *P < 0.05, **P < 0.01, and ***P < 0.001; ANOVA Bonferroni *post hoc* comparison in (H,I). (J) Group occupancy plot for control and KD mice is shown. The platform was located in the lower right quadrant prior to the probe trial and is depicted by a dotted white circle. The value for the maximum occupancy is the maximum found in any of the plots.

Given that loss‐of‐function mutations in *TRIAD3* were identified in patients with dementia and related cognitive deficits, we hypothesized that the *in vivo* knockdown of endogenous TRIAD3A in the mouse hippocampus would lead to deficits in learning and memory, one of the hallmarks of dementia. We utilized TRIAD3A‐sh to reduce endogenous TRIAD3A levels as previously described (Mabb *et al*., 2014) and injected lentiviral particles harboring either TRIAD3A shRNA (KD group) or scrambled shRNA (control group) bilaterally into the CA1 region of the mouse hippocampus (Figs 5A and S4A,B). The knockdown of endogenous TRIAD3A was confirmed by immunohistochemistry using a specific antibody against TRIAD3A, and we compared changes in the levels of endogenous TRIAD3A in TRIAD3A shRNA‐ and scrambled shRNA‐transfected pyramidal neurons within the CA1 region (>25% reduction in immunofluorescence upon TRIAD3A shRNA expression, *P* = 0.004, Student's *t*‐test; Fig. S4C,D). The knockdown of endogenous TRIAD3A was further confirmed by comparing the TRIAD3A levels in mouse cortical neurons expressing TRIAD3A shRNA or scrambled shRNA as was shown previously using cultured rat hippocampal neurons (Fig. S4E) (Mabb *et al*., 2014).

Three weeks after lentiviral infusion, the animals were subjected to a battery of behavioral paradigms. In the open‐field (OF) test, we could not observe any difference in the total distance traveled (*F*_1,20 _= 0.16, *P* = 0.69, two‐way ANOVA, KD vs. control), distance traveled in the center (*F*_1,20 _= 0.70, *P* = 0.41, two‐way ANOVA, KD vs. control) or time spent in the center (KD: 40.55 ± s/min; control: 46.74 ± 3.46 s/min; *P* = 0.2892, Student's *t*‐test) between the two groups (Fig. S5A–D).

Next, to test whether there were any differences in spatial learning and memory between the two groups, we employed two relevant behavioral paradigms: the water‐cross maze (WCM) and the Morris water maze (MWM) (Kleinknecht *et al*., 2012; Morris, 1984). The WCM set‐up consists of a water‐filled maze with four arms (N, S, E, and W), with a hidden platform located in one arm (W) (Fig. 5B). Six trials were conducted per day across 4 days to train the mice to locate the hidden platform. Each trial was conducted after the arm located on the opposite side from the start arm (either S or N) was blocked. Interestingly, we observed that the group injected with the TRIAD3A shRNA exhibited reduced accuracy (*F*_1,20 _= 4.676, *P* = 0.043, two‐way ANOVA, KD vs. control; Fig. 5C), indicating that loss of endogenous TRIAD3A resulted in reduced impaired spatial learning compared with the control (Fig. 5C). However, there were no differences in latency (*F*_1,20_ = 2.06, *P* = 0.16, two‐way ANOVA, KD vs. control) or number of wrong platform visits (*F*_1,20_ = 1.49, *P* = 0.23, two‐way ANOVA, TRIAD3A KD vs. control; Fig. 5D,E).

As an alternative but analogous test for spatial learning and memory, we performed the MWM (Morris, 1984). Mice were trained to reach a hidden platform located in one of the quadrants of the circular gray pool filled with water across 5 days (Fig. 5F). On the 6th day, a 60‐second probe trial was conducted without the platform, and the number of entries and time spent in each quadrant was analyzed. We observed a significant difference in the latency to reach the platform during the training, indicative of a learning disparity between control and KD groups (*F*_1,12_ = 4.9534, *P* = 0.046, two‐way ANOVA with ‘trials’ as repeated measures; Fig. 5G). The control group reached the criterion of reaching the platform in less than 20 s for two consecutive days, whereas the KD group did not succeed in reaching this criterion (learning effects over the 5 days for control: *F*_4,155_ = 9.775, *P* < 0.0001, and KD: *F*_4,115_ = 1.149, *P* = 0.337, one‐way ANOVA). For the probe test, which was used to measure the retention of spatial location of a hidden platform, we found a significant difference in the number of entries in the platform quadrant (Platform) when compared to the platform opposite quadrant (Opp) or the platform adjacent quadrants (Adj1 and Adj2) in the control group but not in the KD group (control: 9.12 ± 1.00 for Platform, 1.87 ± 0.39 for Opp, 4.12 ± 0.66 for Adj1, 3.87 ± 0.51 for Adj2, *F*_3,28_ = 20.2, *P* = 0.0001, one‐way ANOVA; KD: 5.33 ± 1.74 for Platform, 1.67 ± 0.61 for Opp, 2.17 ± 0.60 for Adj1, 3.67 ± 0.80 for Adj2, *F*_3,20_ = 2.46, *P* = 0.098, one‐way ANOVA; Fig. 5H). Furthermore, we found a significant difference in the time spent in the Platform vs. Opp only for the control group (control: 20.76 ± 2.30 for Platform, 6.28 ± 1.60 for Opp, 16.95 ± 0.2.74 for Adj1, 13.73 ± 2.92 for Adj2, Platform vs. Opp, *P* = 0.002, Bonferroni *post hoc* comparisons for time between both quadrants, with all four quadrants considered; KD: 14.80 ± 4.69 for Platform, 6.91 ± 2.73 for Opp, 8.03 ± 2.13 for Adj1, 28.85 ± 8.01 for Adj2, Platform vs. Opp, *P* = 1.000, Bonferroni *post hoc* comparisons for time between both quadrants with all four quadrants considered; Fig. 5I), suggesting that the KD group could not remember the location of the platform during the probe test (Fig. 5J). Taken together, these results suggest that endogenous knockdown of TRIAD3A in the CA1 region of the hippocampus leads to deficits in spatial learning and memory in mice.

### The R660C and R694C TRIAD3A variants neither interacted with Arc nor promoted its ubiquitination

**Figure 6:**
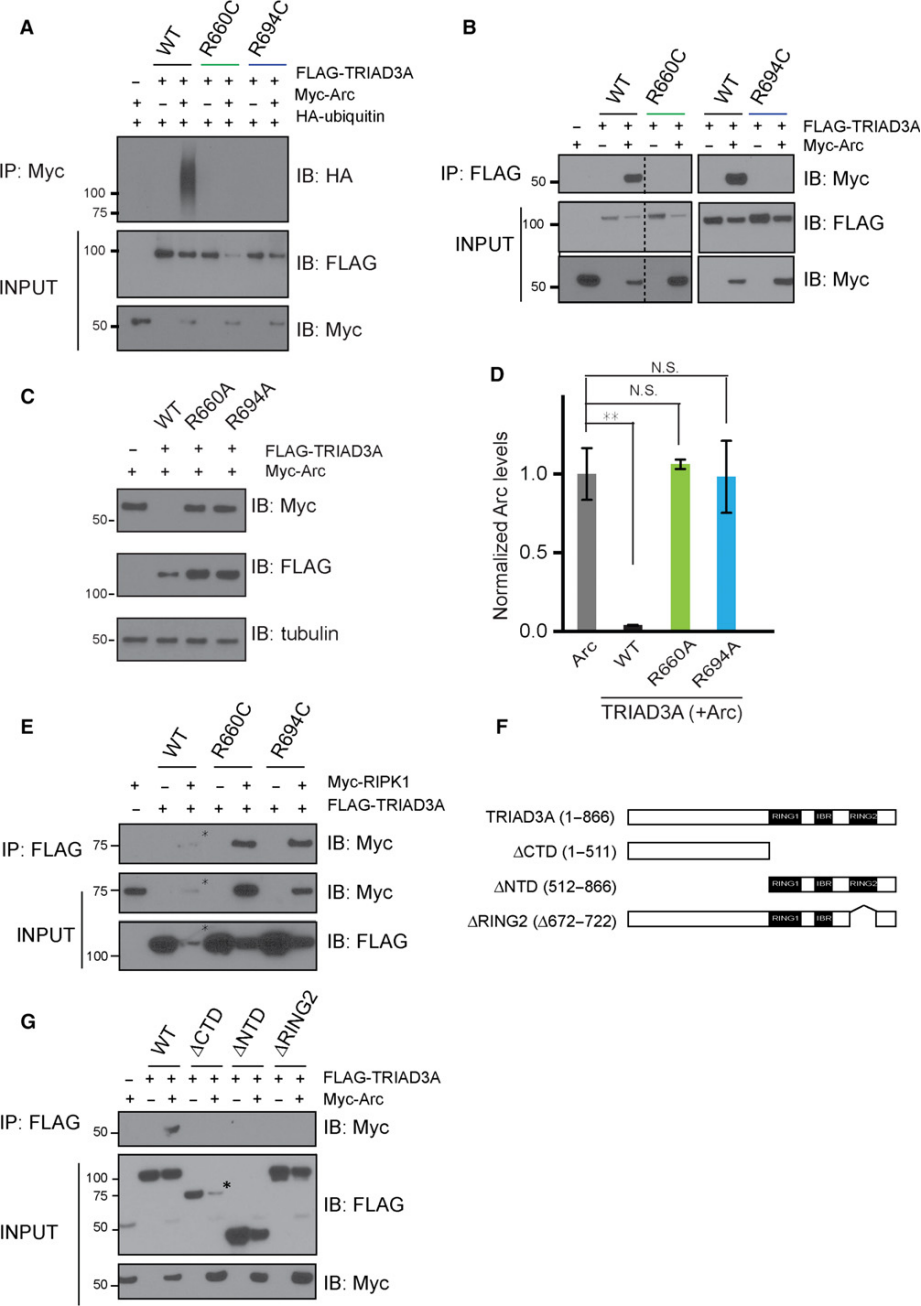
Intact TRIAD3A is required for Arc degradation, ubiquitination, and binding. (A) Ubiquitination assay was performed by transfecting HEK293T cells with Myc‐tagged Arc, HA‐tagged ubiquitin, and FLAG‐tagged TRIAD3A/TRIAD3A variants. The α‐HA (ubiquitin) immunoblot after IP with anti‐Myc beads is shown in the top panel. Only WT TRIAD3A can ubiquitinate Arc; the TRIAD3A variants cannot. The INPUT (2% of entire lysate) samples, which were immunoblotted (IB) with the antibodies indicated, are shown in the bottom two panels. (B) Co‐IP was performed using HEK293T cell lysates cotransfected with Myc‐tagged Arc and FLAG‐tagged TRIAD3A/TRIAD3A variants. IP was performed using α‐FLAG beads and then IB with α‐Myc to probe for Arc pull‐down. The INPUT (2% of entire lysate) samples show the expression of Arc and TRIAD3A in the lysate before IP. The dotted line indicates that the samples were from the same gel, but the lanes were noncontiguous. (C) Top panel: HEK293T cells were cotransfected with 0.25 μg of Arc and 1 μg of WT TRIAD3A and TRIAD3A alanine variants as indicated. Bottom panel: Quantification of Arc levels upon TRIAD3A WT or variants overexpression in HEK293T cells. The data represent the mean ± SEM. Statistical significance was assessed by one‐way ANOVA and Student's *t*‐test (**P < 0.01), *n* = 3. (D) Co‐IP was performed using HEK293T cell lysates cotransfected with Myc‐tagged RIPK1 and FLAG‐tagged TRIAD3A/TRIAD3A variants. IP was performed using α‐FLAG beads and then IB with α‐Myc to probe for RIPK1 pull‐down. RIPK1 binds to WT TRIAD3A and TRIAD3A variants. The INPUT (2% of entire lysate) samples show the expression of RIPK1 and TRIAD3A in the lysate before IP. (E) Schematic of the TRIAD3A protein indicating the truncations that were generated. (F) Co‐IP was performed using HEK293T cell lysates cotransfected with Myc‐tagged Arc and FLAG‐tagged TRIAD3A/TRIAD3A truncated constructs. IP was performed using α‐FLAG beads and then IB with α‐Myc to probe for Arc pull‐down. The INPUT (2% of entire lysate) samples show the expression of Arc and TRIAD3A in the lysate before IP. (*) indicates lower protein levels.

Given that the dementia‐associated TRIAD3A variants could not degrade Arc, we sought to investigate whether the mutations in *TRIAD3* could affect Arc ubiquitination by performing an *in vivo* ubiquitination assay (Mabb *et al*., 2014). The cotransfection of Myc‐tagged Arc with HA(hemagglutinin)‐tagged ubiquitin cDNAs in HEK293T cells resulted in robust Arc ubiquitination in the presence of TRIAD3A WT (Fig. 6A). In contrast, the expression of either TRIAD3A R660C or R694C variants failed to promote Arc ubiquitination as evidenced by the Western blot of HA‐ubiquitin (Fig. 6A). The lack of Arc ubiquitination by either R660C or R694C prompted us to test Arc interaction by performing co‐immunoprecipitation (Co‐IP) experiments in HEK293T cells. Intriguingly, TRIAD3A WT co‐immunoprecipitated with Arc but neither the R660C nor R694C variant co‐immunoprecipitated with Arc (Fig. 6B).

Arginine is the most commonly mutated residue in disease‐causing mutations found in humans (Cooper *et al*., 1998; Krawczak *et al*., 1998) and is commonly exchanged to cysteine, which potentially disrupts protein function by forming disulfide bridges between two cysteine residues within the protein (Thornton, 1981). However, we found that arginine to alanine substitutions in the TRIAD3A variants (R660A and R694A) disrupted Arc binding, ubiquitination, and degradation equally (Figs 6C and S6A,B), indicating that these two residues (R660 and R694) are indeed critical for Arc interaction.

Similar to other known E3 ubiquitin ligases, TRIAD3A has multiple protein‐binding partners in addition to Arc. To investigate whether TRIAD3A R660C and R694C variants have specific synaptic defects due to their lack of binding to Arc selectively, we performed Co‐IP experiments by expressing TRIAD3A WT or variants with the TRIAD3A binding partner RIPK1 [receptor (TNFRSF)‐interacting serine‐threonine kinase 1]. We found that both TRIAD3A R660C and R694C variants bind RIPK1 (Fig. 6D). This suggests that *TRIAD3* mutations are selectively defective in their Arc interaction.

The loss of Arc interaction by substitutions at R660 and R694 residues that lie within the C‐terminal RBR domain of TRIAD3A is puzzling because the C‐terminal domain is quite distant from the N‐terminal region of TRIAD3A (201–470 aa), which was previously shown to be required for Arc binding (Mabb *et al*., 2014). Therefore, we sought to identify Arc interaction domains of TRIAD3A by generating multiple domain‐truncated mutants of TRIAD3A (ΔCTD: 1–511 aa; ΔNTD: 512–866 aa; and ΔRING2: 672–722 aa) and performing Co‐IP assays with Myc‐tagged Arc (Fig. 6E). All mutants failed to interact with Arc, suggesting that the native structure of TRIAD3A is required for Arc interaction (Fig. 6F).

### Missense variants of TRIAD3A did not colocalize with clathrin‐mediated endocytic sites

Arc interacts with endocytic proteins, such as dynamin‐2 and endophilin‐3, to mediate AMPA receptor endocytosis (Chowdhury *et al*., 2006). Previously, it was shown that a pool of Arc proteins localize at clathrin‐coated pits where TRIAD3A resides for subsequent Arc ubiquitination and degradation (Chowdhury *et al*., 2006; Mabb *et al*., 2014). To test whether *TRIAD3A* R660C or R694C mutations affect the subcellular localization of TRIAD3A, we co‐expressed GFP‐tagged TRIAD3A with DsRed‐tagged clathrin light chain (LC) in COS7 cells and examined the colocalization of these proteins at the plasma membrane using total internal reflection fluorescence microscopy (TIRFM). TIRFM revealed punctate spots of GFP‐tagged TRIAD3A that colocalized with clathrin‐coated pits (Fig. S7A). In contrast, TRIAD3A R660C and R694C variants did not colocalize with clathrin puncta (Fig. S7A). In neurons, TRIAD3A WT puncta colocalized with clathrin‐DsRed signals in dendritic shafts (percent of puncta colocalized with clathrin signal, 68.66 ± 6.98%, *n* = 9; Fig. S7B,C). However, both TRIAD3A R660C and R694C variants exhibited diffused patterns within neuronal dendrites and did not colocalize with clathrin puncta (percent of puncta colocalized with clathrin signal, R660C: 25.75 ± 2.98%, *P* = 0.00002, *n* = 11; R694C: 30.89 ± 5.99%, *P* = 0.00018, *n* = 9, compared with WT; Fig. S7B,C). These results demonstrate that missense variants of TRIAD3A (R660C and R694C) not only affect interaction with Arc but also decrease the subcellular localization of TRIAD3A at endocytic sites, where TRIAD3A functions to regulate Arc turnover in neurons.

## Discussion

We demonstrated that misregulated Arc levels resulting from TRIAD3A loss‐of‐function variants disrupted surface AMPA receptors and basal synaptic transmission. Furthermore, intra‐CA1 delivery of shRNA against TRIAD3A in mouse *in vivo* resulted in deficits in spatial learning and memory (Fig. 5). We showed that the two GHS‐related *TRIAD3A* variants (R660C and R694C) failed to bind and ubiquitinate Arc, did not localize at the endocytic sites and could not regulate Arc levels as a consequence (Figs 6 and S7). Considering these findings, we propose that TRIAD3A loss of function and consequent protein homeostasis failure result in synaptic dysfunction that underlies cognitive deficits in dementia patients.

### Arc misregulation links synaptic deficits with UPS dysfunction as the basis for cognitive decline in dementia

Among the types of dementias, AD appears to be a disorder of synaptic failure, which involves disruptions in synaptic structure and function that consequently lead to aberrant neural processing and cognitive behavioral deficits (DeKosky *et al*., 1996). However, how does Arc misregulation lead to cognitive deficits? Upon activation, Arc is targeted to the dendritic spines and shafts of excitatory neurons, where it interacts with endophilin 2/3 and dynamin to facilitate AMPA receptor endocytosis. This Arc‐mediated AMPA receptor endocytosis decreases the activity of neuronal networks as well as spine size and types (Peebles *et al*., 2010). If Arc‐mediated endocytosis remains unchecked, then excessive modifications of synaptic strength might generate instability or altered synchrony in neuronal networks, subsequently leading to disease states characterized by network imbalances, as observed in AD.

Arc gain of function has been implicated in synaptic changes in neurodevelopmental and neurodegenerative diseases (Greer *et al*., 2010; Wu *et al*., 2011). Arc interacts with presenilin and confers activity‐dependent increases in γ‐secretase cleavage of amyloid precursor protein to generate Aβ amyloid (Wu *et al*., 2011). Moreover, elevated Arc protein expression was observed in the gray matter of the medial frontal cortices of patients with AD (Wu *et al*., 2011) and of certain AD mouse models (Lacor *et al*., 2004; Rosi *et al*., 2005; Perez‐Cruz *et al*., 2011). The exact underlying mechanisms by which Arc is upregulated in AD are unknown; however, these findings indicate that the Arc protein might be an important molecular determinant in regulating synaptic function in the brain.

UPS malfunction has been reported in a host of neurodegenerative disorders such as AD, Parkinson's disease, amyotrophic lateral sclerosis, and Huntington's disease (Keller *et al*., 2000; McNaught *et al*., 2003; Seo *et al*., 2004). Previous studies have shown that protein degradation by the UPS is critical for the retrieval of contextual fear memories during memory retrieval tests by polyubiquitination of synaptic activity‐regulated proteins such as Shank and guanylate kinase‐associated protein, and for the formation of long‐term memory in inhibitory avoidance tests (Lopez‐Salon *et al*., 2001; Lee *et al*., 2008). Moreover, many ubiquitin E3 ligases, such as STUB1, Parkin, and Ube3A, have been implicated in several neurodegenerative disorders, suggesting a link between ubiquitination and neurodegeneration.

Dementia observed in the patients with GHS exhibits characteristics similar to other types of age‐dependent dementing illnesses: (i) neuropathological analysis on the postmortem brain tissue of a GHS patient with dementia‐harboring *TRIAD3* mutations (Margolin *et al*., 2013) revealed the presence of neuronal ubiquitin‐positive inclusions observed in patients with frontotemporal dementia (Rosso *et al*., 2001; Weder *et al*., 2007) and (ii) mutations in *TRIAD3* were recently identified in patients suffering from Huntington‐like disease (HDL) with various symptoms including progressive dementia. Interestingly, a parent of two siblings suffering from HDL with heterozygous *TRIAD3* mutation, was diagnosed with late‐onset dementia and Parkisonism. (Santens *et al*., 2015). Elevated Arc levels due to TRIAD3A knockdown that results in lower basal synaptic transmission might impair synaptic plasticity and subsequently lead to memory loss in patients with dementia. The spatial memory deficits exhibited by mice due to hippocampal TRIAD3A knockdown mirrors the memory impairment observed in patients with dementia. Our current findings of Arc misregulation by loss‐of‐function TRIAD3A variants found in patients with GHS indicate that these two pathways converge as the molecular mechanism underlying dementia in these patients.

### Identification of TRIAD3 variants in dementia and other neurodegenerative diseases

The GHS patients with *TRIAD3* mutations described by Margolin *et al*. also exhibited early‐onset ataxia and hypogonadotropism. Could TRIAD3 variants be found in other neurodegenerative disorders with overlapping symptoms? More recent studies have reported multiple mutations in *TRIAD3* in patients suffering from pediatric‐onset ataxia (R686X), 4H syndrome, and Huntington‐like disorder (Q302X, G456E, and Y539C) (Sawyer *et al*., 2014; Ganos *et al*., 2015; Santens *et al*., 2015). Intriguingly, although the clinical characteristics between patients with GHS and HDL were varied, cerebellar ataxia, dementia, and cognitive defects were some of the common phenotypes in all of these studies with *TRIAD3* mutations (Sawyer *et al*., 2014; Santens *et al*., 2015). If possible, measuring the TRIAD3A levels in the brains of these patients to test for a decrease in TRIAD3A would be extremely informative. We suggest that Arc misregulation may underlie cognitive deficits in these patients.

Although we hypothesized the role of TRIAD3A in dementia, does the loss of function of TRIAD3 lead to cerebellar ataxia, hypogonadotropism comorbid in patients with dementia? Experimental evidence demonstrated that knockdown of the TRIAD3 homolog in zebrafish by morpholino induced cerebellar disorganization, which could not be rescued by co‐expression of the R694C variant (Margolin *et al*., 2013). Moreover, Arc is reported to be required for synapse refinement in the developing mouse cerebellum, which potentially affects cerebellar function in controlling motor coordination (Mikuni *et al*., 2013). Given that TRIAD3A is expressed moderately in the cerebellum, testing whether the cerebellar‐specific ablation of TRIAD3A or cerebellar‐specific overexpression of Arc using conditional knockout or transgenic mice, respectively, can trigger defective motor behaviors or ataxia will be interesting. Moreover, the TRIAD3A variants have been proposed to affect gonadotropin‐releasing hormone (GnRH) secretion from the hypothalamus by the continuous activation and maintenance of NF‐κB signaling, resulting in hypogonadism (Chen *et al*., 2002; Miah *et al*., 2011; Margolin *et al*., 2013; Zhang *et al*., 2013).

In conclusion, the functional validation of TRIAD3A variants found in patients with dementia suggests that the deficits in Arc modulation and basal synaptic transmission potentially lead to cognitive decline.

## Experimental procedures

Detailed information regarding electrophysiology, immunocytochemistry, co‐immunoprecipitation, *in vivo* ubiquitin assays, TIRFM, image acquisition, and animal behavior are included in Data S1 (Supporting information).

### DNA and shRNA constructs

Mutations corresponding to C540X, R660C, and R694C were introduced into the pEGFP‐C3 TRIAD3A construct by site‐directed mutagenesis (Mabb *et al*., 2014), and into the pRK5‐FLAG TRIAD3A (human) construct, which was obtained from Dr. Tsung‐Hsien Chuang (Sanford‐Burnham Medical Research Institute). The corresponding R660A and R694A mutations were generated in the pRK5‐FLAG TRIAD3A construct. The TRIAD3A ΔCTD (1‐511), Δ(1‐ (512‐866), Δ(512‐ ((512‐866), and TRIAD3A‐R constructs were generated using pRK5‐FLAG TRIAD3A as a template. The Myc‐Arc and clathrin‐DsRed constructs were gifts from Dr. Paul Worley (Johns Hopkins University) (Chowdhury *et al*., 2006) and Dr. Jim Keen (Thomas Jefferson University), respectively. HA‐ubiquitin was a gift from Dr. Kah Leong Lim (National University of Singapore). All the mutations and truncations were verified by DNA sequencing. TRIAD3A‐sh and Scr lentiviral constructs (pLentilox 3.7) were generated as reported previously (Mabb *et al*., 2014).

### Neuronal cell culture

Cortical neuronal cultures were prepared by dissecting out the cortices of E18 rat or E16 mouse embryos (SD rats, C57Bl6 mice, InVivos, Singapore). The cortices were dissociated and plated on poly‐L‐lysine (Sigma, St. Louis, Missouri, United States)‐coated 18‐mm coverslips at the desired density. These neurons were cultured in neurobasal medium (Invitrogen) containing B27 supplement (Invitrogen, Waltham, Massachusetts, United States), GlutaMAX (Invitrogen), and penicillin–streptomycin (Invitrogen). In addition, 5‐fluoro‐2′‐deoxyuridine (Sigma) was added to prevent glial proliferation. The neurons were transfected using a calcium phosphate transfection kit (Clontech, Mountain View, California, United States) according to the manufacturer's instructions. All procedures with rats were conducted in accordance with the protocol approved by the Institutional Animal Care and Use Committee (IACUC) of the Duke‐NUS Graduate Medical School. Human embryonic kidney (HEK293T) cells were cultured in DMEM (Gibco) containing 10% fetal bovine serum (Gibco, Waltham, Massachusetts, United States) and penicillin–streptomycin.

### Animal surgery

All studies were conducted with protocols approved by the Institutional Animal Care and Use Committee (IACUC) of the Duke‐NUS Graduate Medical School.

For behavioral experiments, 5‐week‐old WT (C57Bl6/Ntac) mice were purchased from InVivos and housed in a vivarium for 2 weeks to acclimatize before carrying out stereotaxic surgery. Virus expressing either TRIAD3A‐sh or Scr shRNA was injected bilaterally into the CA1 region of the dorsal hippocampus using two coordinates: (i) AP + 1.5, ML ± 0.9, DV −2.0; and (ii) AP + 2.5, ML ± 1.9, DV −2.0. The animals were allowed to recover in their home cage for three weeks. The site of stereotaxic injections in all the mice was confirmed after completion of the behavioral experiments. Behavioral experiments were conducted on 3‐ to 6‐month‐old male C57Bl6 mice.

### Statistical analysis

At least three experiments were performed independently under each experimental condition, and similar results were obtained. Statistical analyses were performed using Student's *t*‐test for comparison between two groups. ANOVA was performed to calculate differences among multiple means followed by Sidak's or Bonferroni's multiple comparison *post hoc* test. All data are presented as the mean and standard error of the mean (mean ± SEM). Statistical significance was defined when ****P < 0.0001, ***P < 0.001, **P < 0.01, *P < 0.05 compared with the control.

## Conflict of interest

None declared.

## Funding

This work was supported by the Singapore Ministry of Education (MOE) Academic Research Fund (MOE2014‐T2‐2‐071 to H.S.J.), National Medical Research Councel Collaborative Research Grant (NMRC/CBRG/0075/2014 to H.S.J.), and a Duke‐NUS Signature Research Program Block Grant (to H.S.J.), the SingHealth Foundation (# SHF/FG504P/2012 to Z.B.), an NNI Centre Grant (# NCG BF04 to Z.B.), a Johns Hopkins Alzheimer's Disease Research Center Grant (NIH P50AG05146 to O.P.), and a Khoo Postdoctoral Fellowship Award (Duke‐NUS‐KPFA/2015/0001 to N.H.).

## Author contributions

N.H. designed and performed the biochemical experiments, stereotaxic viral injection, behavioral assays, and immunohistochemistry; Q.Y. contributed to ideas and designed and performed most of the experiments especially electrophysiological studies; Y.C.Y. performed behavioral assays and analyzed data; O.P. prepared and provided tissue samples; P.W. provided tissue samples and antibodies, supervised experiments, and edited the manuscript; D.Q.S. and Z.B. performed and analyzed the Morris water maze experiment; H.S.J. contributed to ideas and designed and supervised all experiments; N.H. and H.S.J. wrote the manuscript.

## Supporting information


**Data S1** Extended experimental methods.
**Fig. S1** Overexpression of Arc‐KR restores Arc level when coexpressed with TRIAD3A.
**Fig. S2** Validation of TRIAD3A shRNA and Rescue.
**Fig. S3** The decrease in basal synaptic transmission by TRIAD3A knockdown is due to increased Arc protein levels.
**Fig. S4** Triad3A knockdown in the CA1 region of mouse Hippocampus
**Fig. S5** Open field test.
**Fig. S6** TRIAD3A alanine variants neither interacted with Arc not promoted its ubiquitination.
**Fig. S7** TRIAD3A variants were not colocalized with clathrincoated pits.
**Fig. S8** Original western blot images for Figure 6B.
**Fig. S9** Original western blot images for Figure S6A.
**Table S1** Results of statistical analyses.
