# Supplementary material for: TRIAD3/RNF216 mutations associated with Gordon Holmes syndrome lead to synaptic and cognitive impairments via Arc misregulation
Source: Aging Cell. 2016 Dec 20;16(2):281–92. doi: 10.1111/acel.12551 (PMC5334534; doi:10.1111/acel.12551)
Supplement: Supplementary file 1 — Data S1 Extended experimental methods. Fig. S1 Overexpression of Arc‐KR restores Arc level when coexpressed with TRIAD3A. Fig. S2 Validation of TRIAD3A shRNA and Rescue. Fig. S3 The decrease in basal synaptic transmission by TRIAD3A knockdown is due to increased Arc protein levels. Fig. S4 Triad3A knockdown in the CA1 region of mouse Hippocampus Fig. S5 Open field test. Fig. S6 TRIAD3A alanine variants neither interacted with Arc not promoted its ubiquitination. Fig. S7 TRIAD3A variants were not colocalized with clathrincoated pits. Fig. S8 Original western blot images for Figure 6B. Fig. S9 Original western blot images for Figure S6A. Table S1 Results of statistical analyses. [file ACEL-16-281-s001.pdf]

Extended Experimental Methods:

Immunohistochemistry

Mouse brain slices were permeabilized, blocked and immunostained with primary antibodies specific for GFP or Triad3A. For surface GluA1 staining, cultured cortical neurons were subjected to live antibody feeding for 10 minutes at room temperature with anti-GluA1 antibody (a gift from Dr. Paul Worley, 1:100 dilution) as described previously (Mabb *et al.* 2014). Then, the neurons were washed twice with PBS, fixed in a 4% PFA solution of PBS containing 4% sucrose, blocked, and incubated with an Alexa dye-conjugated secondary antibody (Life Technologies) for 1 hour at room temperature before mounting on microscope slides.

Image acquisition, analysis, and quantification

All images were obtained with either a 10x, 40x or 63x oil objective on an LSM 710 (Zeiss) confocal microscope. Images used for quantification were acquired using the same parameters and analyzed using MetaMorph (Universal Imaging) software. For surface GluA1 quantification, the GFP channel was binarized and used as a mask. The GluA1 channel was superimposed onto the GFP channel using the "AND" function. The same threshold was used for all neurons. The fluorescence intensity for a selected region of the dendrite was measured using the "integrated morphometry analyses" function in MetaMorph and divided by the dendrite length of the selected region. For colocalization analyses, both the GFP and clathrin channels were binarized and thresholded, and colocalization was measured using the "colocalization" application in MetaMorph.

Total internal reflection fluorescence microscopy (TIRFM)

For TIRFM image acquisition, COS7 cells were plated onto glass-bottomed culture dishes (World Precision Instruments # FD35-100) and transfected with Lipofectamine 2000 (Axelrod 1981; Thompson *et al.* 1981; Merrifield *et al.* 2005; Mabb *et al.* 2014). Imaging was performed in Tyrode's solution (139 mM NaCl, 3 mM KCl, 17 mM $NaHCO_3$, 12 mM glucose, 3 mM $CaCl_2$, and 1 mM $MgCl_2$), and cells were maintained at 37°C during the experiments. The TIRFM setup consisted of a Nikon Eclipse Ti inverted microscope (Nikon) equipped with an EMCCD camera (Andor Technology), 100x oil immersion objective (1.45 NA) and argon (488 nm) and argon/krypton (568 nm) lasers. Focal drift was prevented by an in-built autofocus assist system.

Electrophysiology

Whole-cell voltage clamp recordings were performed to record mEPSCs from transfected neurons (Zhang & Trussell 1994). The extracellular solution contained (in mM) 124 NaCl, 3 KCl, 1.3 NaH_2PO_4, 10 dextrose, 2 $MgCl_2$, 2 $CaCl_2$, 10 HEPES, 0.05 bicuculline, 0.05 amino-5-phosphonovaleric acid (APV) and 0.001 tetrodotoxin (TTX) at pH 7.4. Recording pipettes with resistances of 4–6 MΩ were filled with an internal solution containing (in mM) 120 K-gluconate, 9 KCl, 10 KOH, 3.48 $MgCl_2$, 4 NaCl, 10 HEPES, 4 Na_2ATP, 0.4 Na_3GTP, 17.5 sucrose, and 0.5 EGTA with an osmolarity of 290 mOsm and pH 7.3. Neurons were held at −70 mV using an Axon MultiClamp 700B amplifier (Axon Instruments). The signal was sampled at 40 kHz and filtered at 2 kHz (DigiData 1440A, Molecular Devices). Cells with a capacity lower than 40 pF or serial resistance higher than 20 M0 kHz and filtered at the analysis. The data were analyzed offline using the ClampFit (Molecular Devices) or

Mini Analysis (Synaptosoft) program. The miniature events were sorted out by series of parameters including: amplitude(>6 pA), current charged area (>15) (Figure R7). All data were auto-sorted followed by manually removing false events with wrong trace profile. The rise and decay kinetics were comparable across analysed groups.

Co-immunoprecipitation (Co-IP)

The FLAG-TRIAD3A-WT, FLAG-TRIAD3A-R660C or FLAG-TRIAD3A-R660C variant was co-transfected into HEK293T cells with Myc-Arc or Myc RIPK1 using Lipofectamine 2000. At 48 hours post-transfection, lysates were prepared using Co-IP buffer (20 mM Tris-HCl, 3 mM EDTA, 3 mM EGTA, 150 mM NaCl, 1% Triton X-100 [pH 7.4], and 1 mM PMSF) supplemented with protease inhibitors (Roche #05892791001) and a phosphatase inhibitor (Sigma #P5726). Pre-clearing of the lysates was performed with IgG agarose (Sigma #A0919) for 1 hour at 4°C with constant rotation. TRIAD3A or the TRIAD3A variants were immunoprecipitated by overnight incubation with anti-FLAG beads (Sigma #A220). The samples were washed thrice with Co-IP buffer, and the proteins were eluted by boiling the beads with LDS sample buffer (Life Technologies #NP0007). The immunoprecipitated proteins were resolved by gel electrophoresis, transferred to a PVDF membrane and probed for Arc and TRIAD3A proteins using anti-Myc (Millipore #06-549) and anti-FLAG (Sigma #F3165) antibodies, respectively.

***In vivo* ubiquitination assay**

The *in vivo* ubiquitination assay was performed as described previously (Mabb *et al.* 2014). Briefly, HEK293T cells transfected with the FLAG-TRIAD3A-WT, FLAG-TRIAD3A-R660C, or FLAG-TRIAD3A-R660C constructs were transfected into

HEK293T cells along with Myc-Arc and HA-ubiquitin. The cell lysates were prepared the next day using IP buffer containing 1% SDS and subsequently boiled for 20 minutes. The cell debris was removed by centrifugation, and the samples were diluted 10 times with IP buffer containing phosphatase and protease inhibitors, 10 mM N-ethylmaleimide, and 3 mM iodoacetamide so that the final SDS concentration was 0.1%. Arc was immunoprecipitated using anti-Myc beads (Sigma #A7470), washed 3 times, and subjected to western blot following gel electrophoresis. The membrane was probed with anti-HA antibody (Covance #MMS-101R) to detect Arc ubiquitination.

Behavioral procedures

Open field (OF) test: For OF experiments, the animals were placed in the center of an acrylic animal cage for 1 hour, and their activity was assessed and calculated every 5 minutes using a VersaMax activity monitoring system.

Water-cross maze (WCM): The WCM was constructed in-house at DUKE-NUS and the experiment was conducted as described previously (Kleinknecht *et al.* 2012). The WCM arena consists of a water-filled maze with 4 arms (north [N], south [S], east [E] and west [W]) with the platform located in one arm (W). The platform was placed in the west arm of the water-filled maze, and 6 trials were performed each day for 4 days for the animal to learn the location of the platform. Each trial was conducted after blocking the arm opposite to the start arm (S or N), effectively mimicking a T-maze. Among the 6 trials carried out each day, 3 trials were started from the south arm, and the remaining three were initiated from the north arm in a randomized order. During each trial, the animal was given 30 seconds to locate the platform, after which it was gently prodded to the platform. The time taken to reach

the platform *viz* latency, the direct navigation from the start arm (S or N) to the arm containing the platform (W) *viz* accuracy, and the number of visits to half the distance of the arm opposite arm without the platform (E) *viz* wrong platform visits, are considered measures of learning. One or two-way analysis of variance (ANOVA) was used according to the number of factors considered followed by Sidak's *post hoc* comparisons where applicable.

Morris water maze (MWM): The water maze consisted of a 120 cm diameter grey circular pool filled with water (23–26°C, 40 cm deep) made opaque by adding non-toxic white paint (Crayola). The pool was surrounded by several distant cues from the environment of the experimental room. The animals learned to find a transparent platform (10 cm in diameter) hidden 1 cm below the water surface; the location of the platform remained constant throughout the experiment. Each animal was tested four times a day across five consecutive days, with an intertrial of approximately 45 minutes. For each trial, the mice were released facing the tank wall from one randomly selected starting points (N, S, E or W) and were allowed to swim until they reached the platform. Animals that failed to find the platform within 1 minute (60 seconds) were gently directed to it and put on it for 15 seconds. After the trial, the mice were removed from the pool, gently dried with a towel, put individually in cages filled with paper towel, and warmed with water bottles to avoid hypothermia. The criterion of learning success consisted of reaching the platform in less than 20 seconds. On the 6th day, a 60-second probe trial was conducted without platform and the time spent in each quadrant was analyzed. Trials were recorded with a video camera placed above the center of the pool, and the analysis was automated through the ANY-maze video tracking system (Stoelting, USA). Data (means and standard error of the means) were analyzed with IBM SPSS Statistics v19.0 software. One or

two-way ANOVA was used according to the number of factors considered ("treatment", time in "days" or "trials", and "quadrants"), followed by Bonferroni *post hoc* comparisons if needed. Mice that remained immobile for more than 25 seconds in 7 or more trials during the training phase were excluded from the analyses.

References:

Axelrod D (1981). Cell-substrate contacts illuminated by total internal reflection fluorescence. *The Journal of cell biology*. **89**, 141-145.

Kleinknecht KR, Bedenk BT, Kaltwasser SF, Grunecker B, Yen YC, Czisch M, Wotjak CT (2012). Hippocampus-dependent place learning enables spatial flexibility in C57BL6/N mice. *Frontiers in behavioral neuroscience*. **6**, 87.

Mabb AM, Je HS, Wall MJ, Robinson CG, Larsen RS, Qiang Y, Correa SA, Ehlers MD (2014). Triad3A regulates synaptic strength by ubiquitination of Arc. *Neuron*. **82**, 1299-1316.

Merrifield CJ, Perrais D, Zenisek D (2005). Coupling between clathrin-coated-pit invagination, cortactin recruitment, and membrane scission observed in live cells. *Cell*. **121**, 593-606.

Thompson NL, Burghardt TP, Axelrod D (1981). Measuring surface dynamics of biomolecules by total internal reflection fluorescence with photobleaching recovery or correlation spectroscopy. *Biophysical journal*. **33**, 435-454.

Zhang S, Trussell LO (1994). Voltage clamp analysis of excitatory synaptic transmission in the avian nucleus magnocellularis. *The Journal of physiology*. **480 (Pt 1)**, 123-136.

Supplementary Figure Legends.

Supplementary Figure 1. Overexpression of Arc-KR restores Arc level when co-expressed with TRIAD3A.

(A) Immunostaining for Arc was performed on cultured cortical neurons transfected with GFP, TRIAD3A+Arc and TRIAD3A+Arc-KR. **(B)** Arc intensity was normalized to untransfected neurons within each coverslip. All histogram data are shown as the mean ± SEM. Statistical significance was assessed by one- way ANOVA (*$p < 0.05$).

Supplementary Figure 2. Validation of TRIAD3A shRNA and Rescue.

Western blot analysis of shRNA-resistant TRIAD3A (R) or variants (R660C and R694C) co-transfected with Triad3A shRNA (TRIAD3A-sh) or scrambled shRNA (Scr) in HEK293T cells. Tubulin is shown as a loading control.

Supplementary Figure 3. The decrease in basal synaptic transmission by TRIAD3A knockdown is due to increased Arc protein levels.

(A) Representative gap-free recorded trace (left) and averaged mEPSC waveform (right) are shown for TRIAD3A-shRNA (TRIAD3A-sh) co-transfected with scrambled shRNA (Scr), or T3A-sh co-transfected with Arc-shRNA (Arc-sh). In the averaged mEPSC waveform panel, grey traces indicate the overlaid raw traces and the red line indicates the average. **(B, D)** Cumulative plots and **(C, E)** histograms of mEPSC amplitude and frequency for TRIAD3A-sh + Scr ($n = 20$) and TRIAD3A-sh + Arc-sh ($n = 20$). All histogram data are shown as the mean ± SEM. Statistical significance was assessed by Student's t-test (***$p < 0.001$).

Supplementary Figure 4. Triad3A knockdown in the CA1 region of mouse hippocampus

(A) The distribution of the lentivirus from a single injection in the CA1 region of the dorsal hippocampus over several coronal brain sections is shown. Scale bar, 500 μm. **(B)** The dotted box in Figure 5 **(A)** is enlarged. The CA1 neurons infected with Triad3A shRNA (GFP+) can be clearly discerned. Scale bar, 10 μm. **(C)** Representative images of shRNA/Scr CA1 neurons (GFP+) immunostained with TRIAD3A antibody (red). Scale bar, 10 μm. **(D)** The quantification is shown as the mean ± SEM (Student's t-test, $^{**}p < 0.01$). **(E)** Mouse cortical neurons (DIV7) were transduced with scrambled (Scr) or Triad3A shRNAs. The proteins were resolved by SDS-PAGE and immunoblotted with Triad3A and tubulin antibodies. A red arrowhead indicates the band corresponding to Triad3A.

Supplementary Figure 5. Open field test. A comparison between the KD and Control group for the total distance traveled **(A, B),** the center distance **(C)** and center time **(D)** is shown.

Supplementary Figure 6. (A) TRIAD3A alanine variants neither interacted with Arc not promoted its ubiquitination.IP was performed using HEK293T cell lysates co-transfected with Myc-tagged Arc and FLAG-tagged TRIAD3A/TRIAD3A alanine variants. IP was performed using bar, 500 μm.ells. Tubulin is shown as a loading control.est (***p < 0.UT (2% of the entirelysate) samples show the expression of Arc and TRIAD3A in the lysate before IP. The dotted line indicates that the samples were from the same gel but the lanes were non-contiguous**. (B) Ubiquitination assay of Arc with TRIAD3A alanine variants.** A ubiquitination assay was performed by transfecting HEK293T cells with Myc-

tagged Arc, HA-tagged ubiquitin and FLAG-tagged TRIAD3A/TRIAD3A alanine variants. The α-HA (ubiquitin) immunoblot after IP with anti-Myc beads is shown in the top panel. Only WT TRIAD3A, not the TRIAD3A alanine variants, can ubiquitinate Arc. The INPUT (2% of the entire lysate) samples are shown in the below two panels immunoblotted (IB) with the indicated antibodies.

Supplementary Figure 7. TRIAD3A variants were not colocalized with clathrin-coated pits.

(A) TIRFM image of GFP-tagged WT TRIAD3A or TRIAD3A variants and clathrin-LC (DsRed) in COS7 cells. Scale bar, 2 μm. Magnified regions are indicated on the right. Scale bar, 0.5 μm. **(B)** Confocal image of GFP-tagged WT TRIAD3A or TRIAD3A variants and clathrin-DsRed in the dendrites of cortical neurons. Scale bar, 5 μm. Magnified regions are indicated on the right. Scale bar, 0.2 μm. **(C)** Quantification of colocalization percentage of TRIAD3A WT or variants with clathrin are shown as the mean ± SEM. Statistical significance was assessed by one-way ANOVA (****$p < 0.0001$; ***$p < 0.001$).

Supplementary Figure 8. Original western blot images for Figure 6B.

The original full blot corresponding to Figure 6B is shown. The dotted red boxes indicate the bands depicted in the main figure.

Supplementary Figure 9. Original western blot images for Supplementary Figure 6A.

The original full blot corresponding to Supplementary Figure 6A is shown. The dotted red boxes indicate the bands depicted in the main figure.

Supplementary Table 1: Results of statistical analyses.

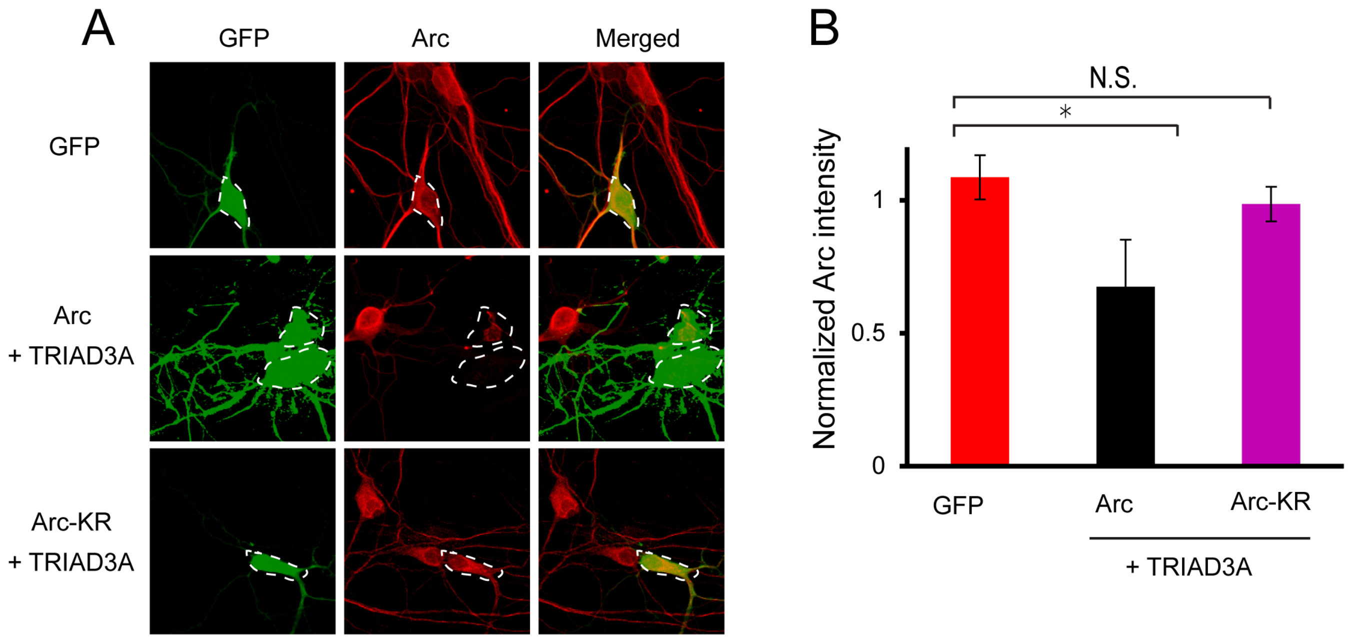

Supplementary Figure 1

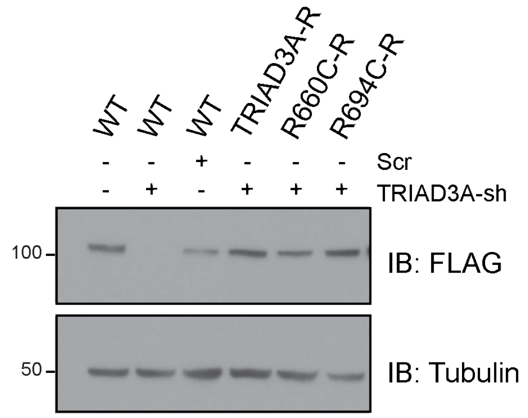

Supplementary Figure 2

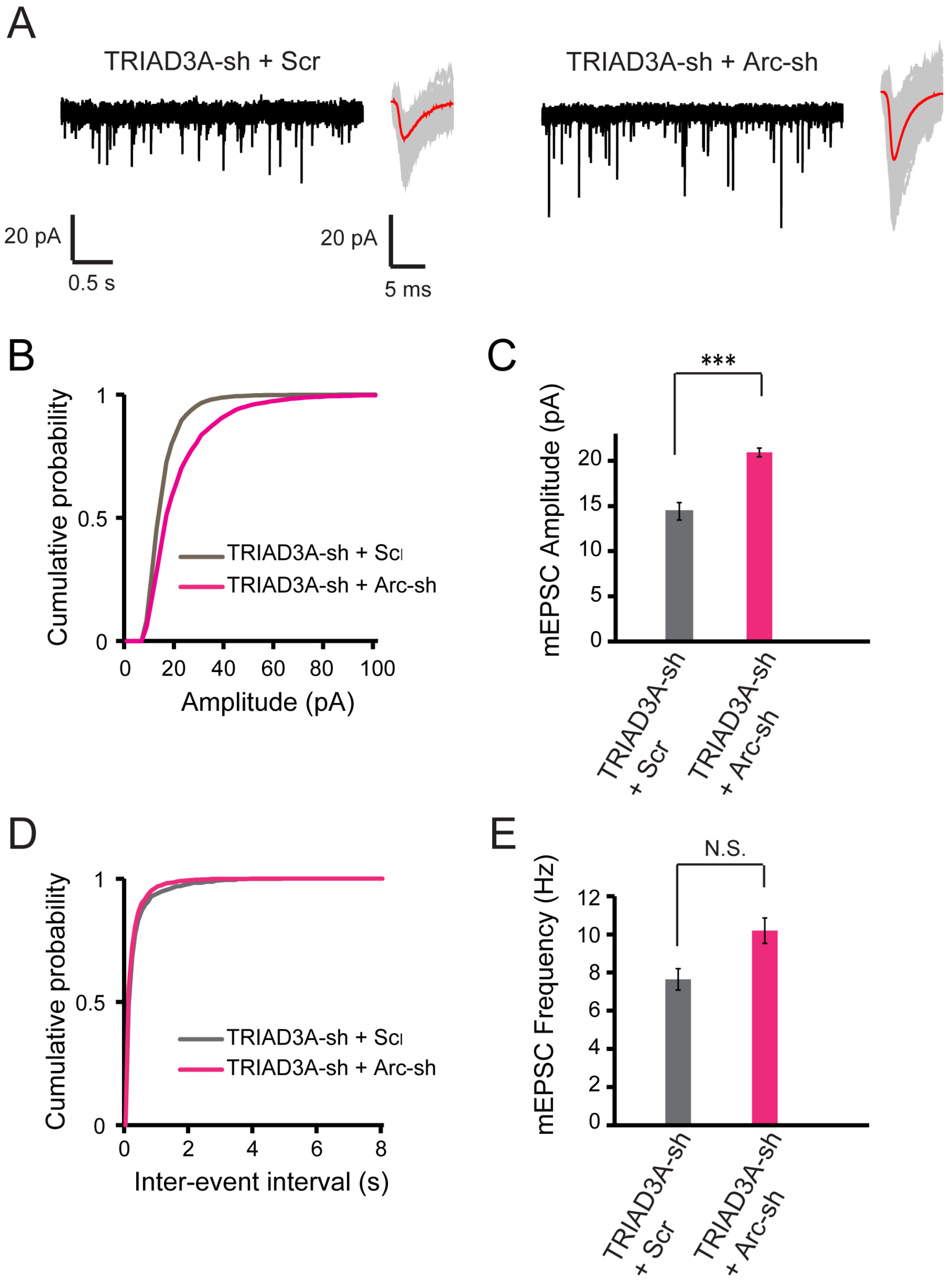

Supplementary Figure 3

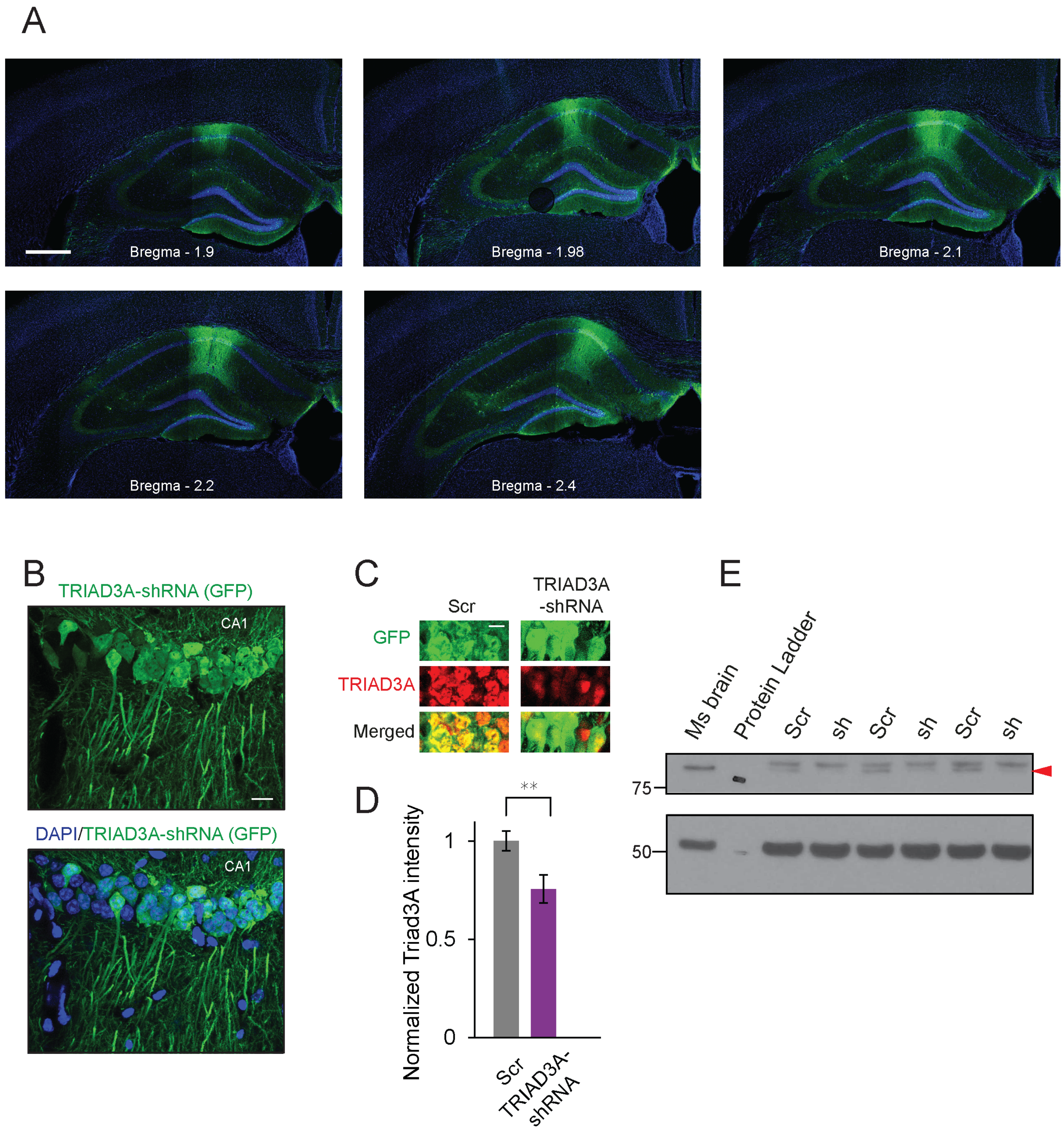

Supplementary Figure 4

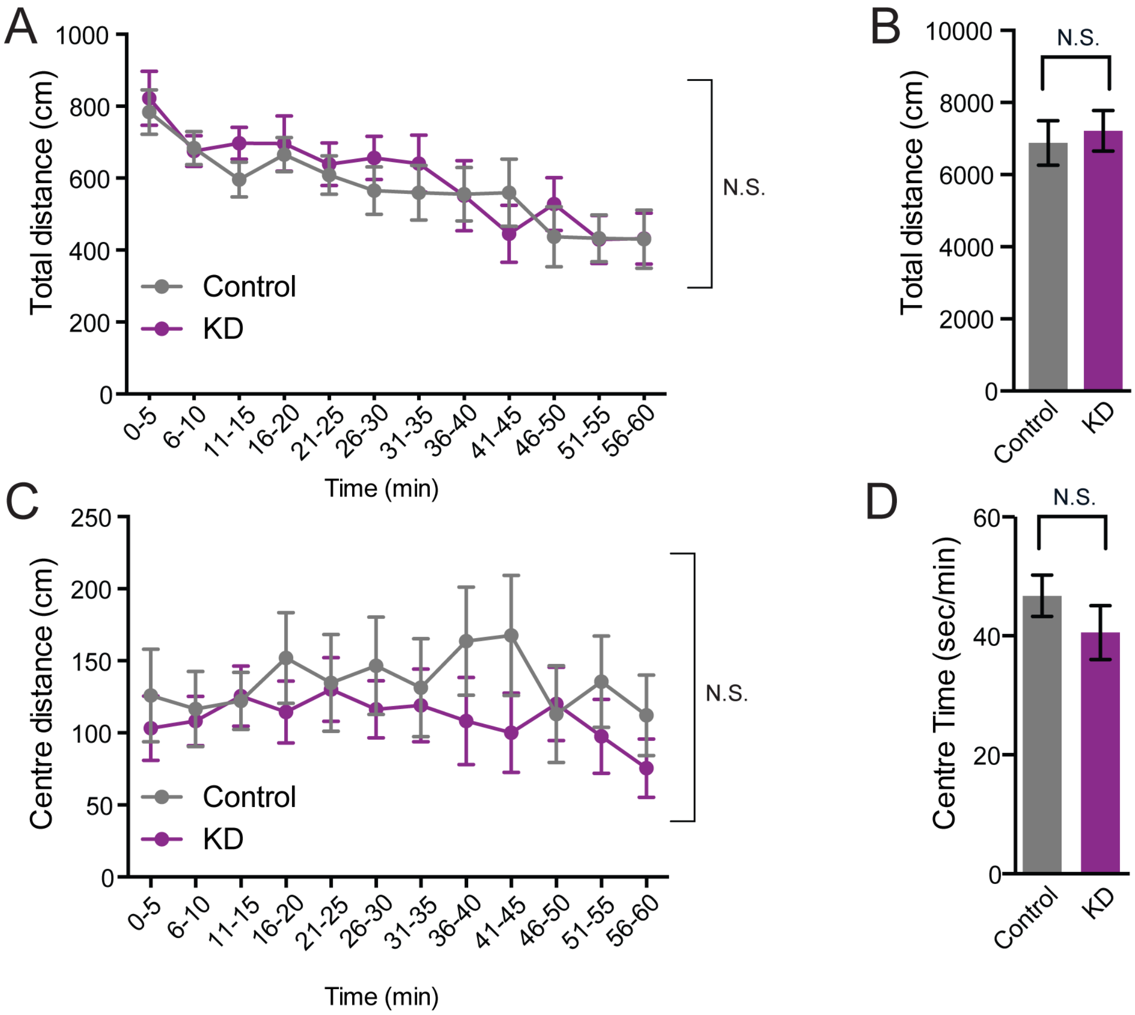

Supplementary Figure 5

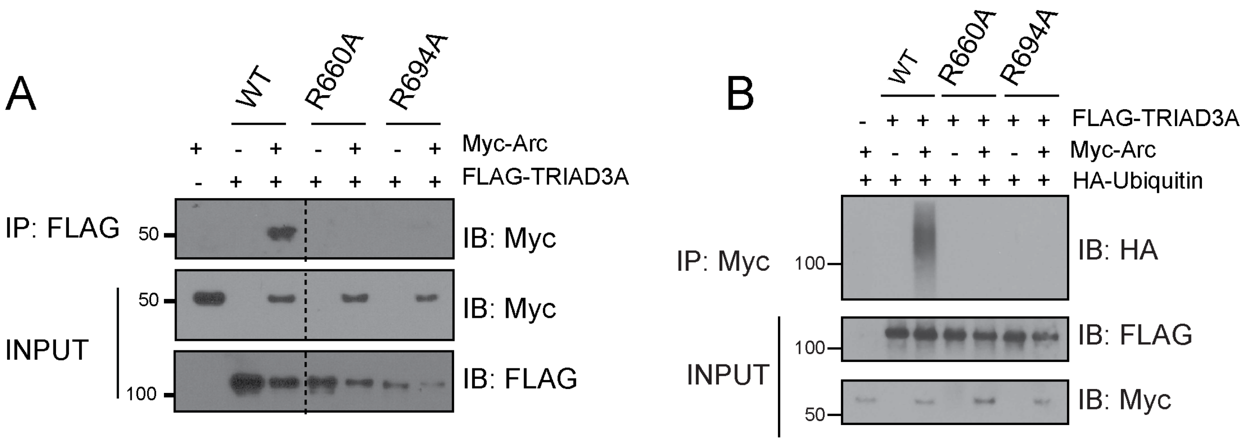

Supplementary Figure 6

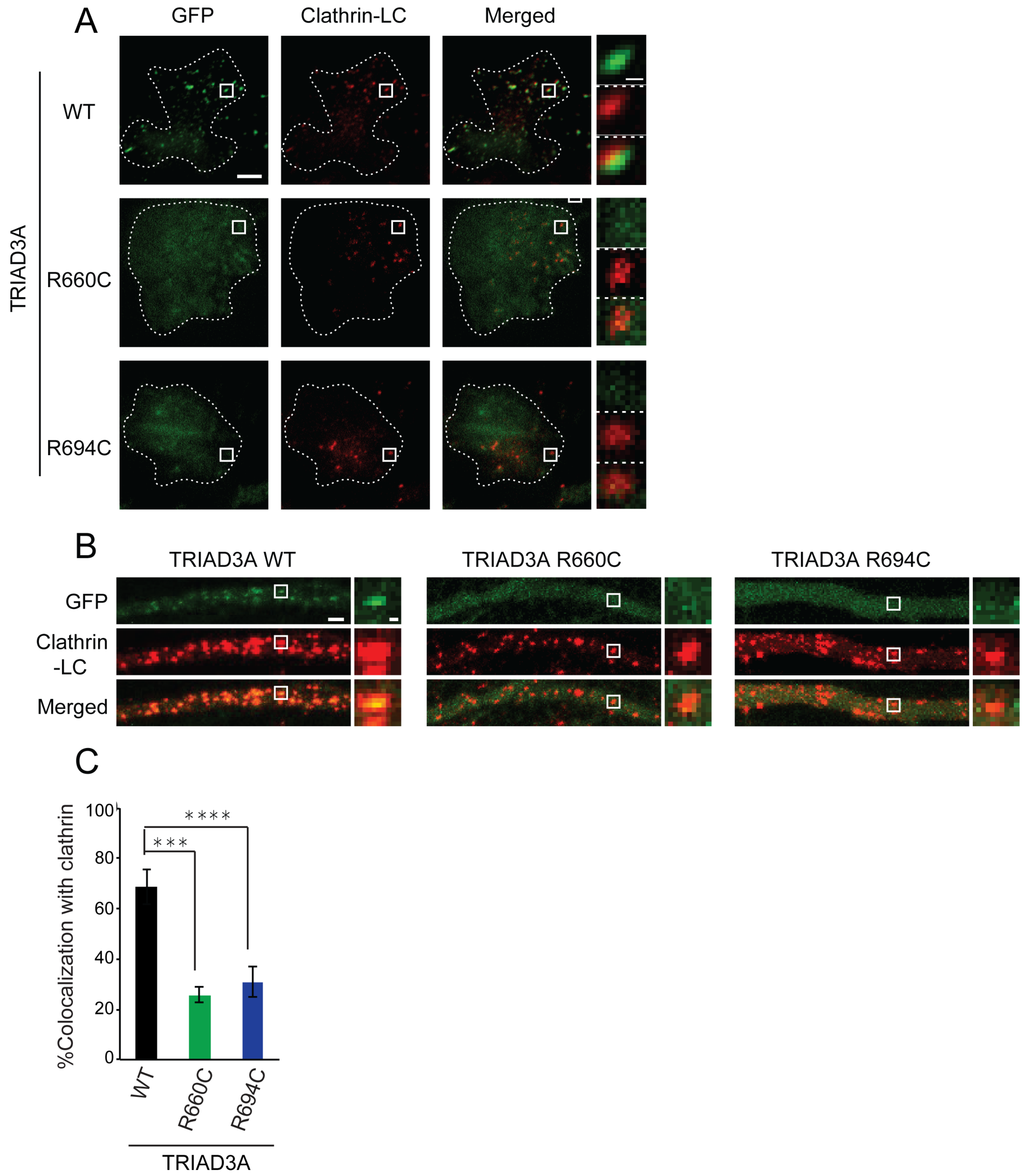

Supplementary Figure 7

Output: anti-Myc

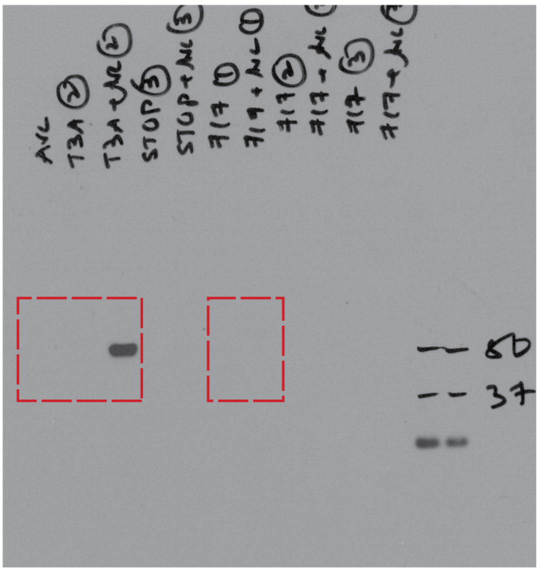

Output: anti-Myc

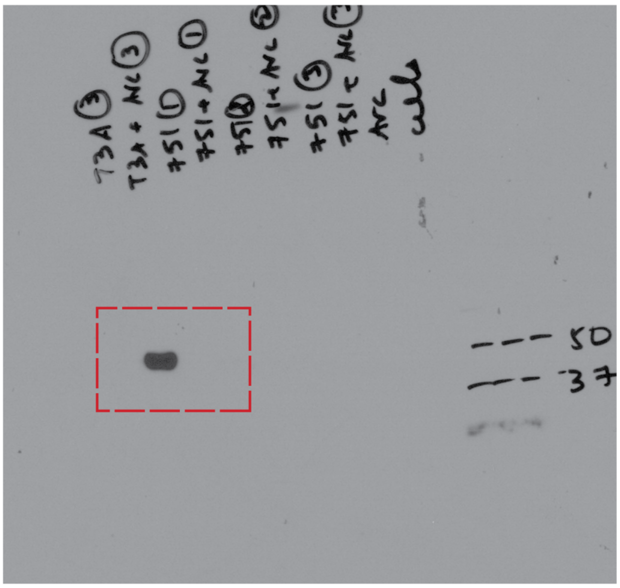

Input: anti-FLAG

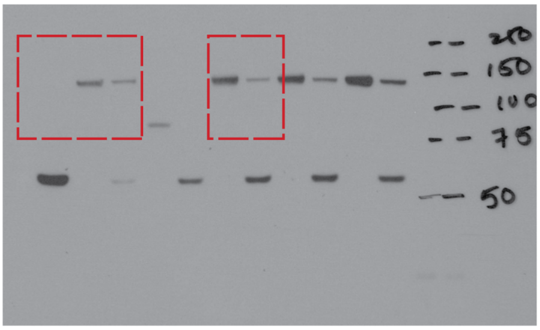

Input: anti-Myc

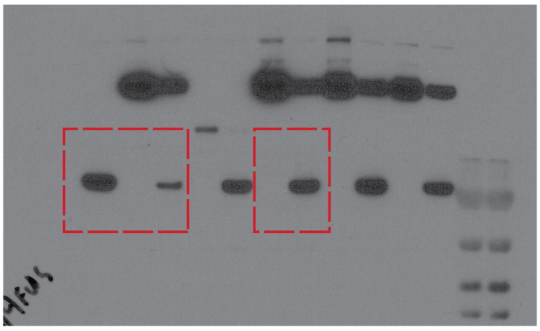

Input: anti-FLAG

Input: anti-Myc

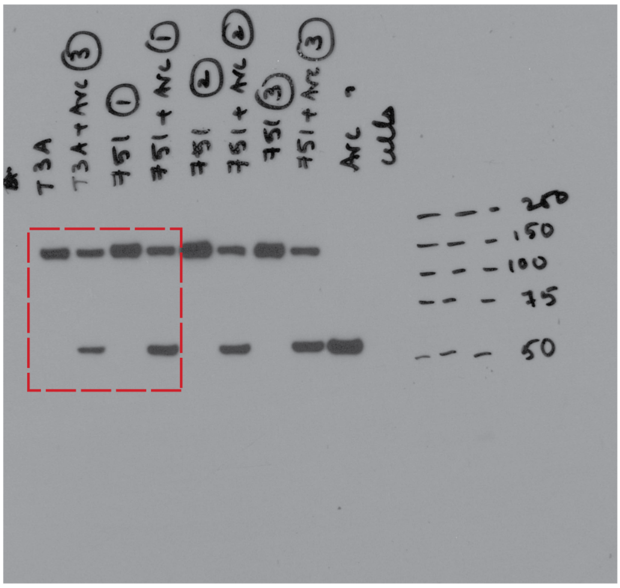

Original blot related to Figure 6B

Supplementary Figure 8

Output: anti-Myc

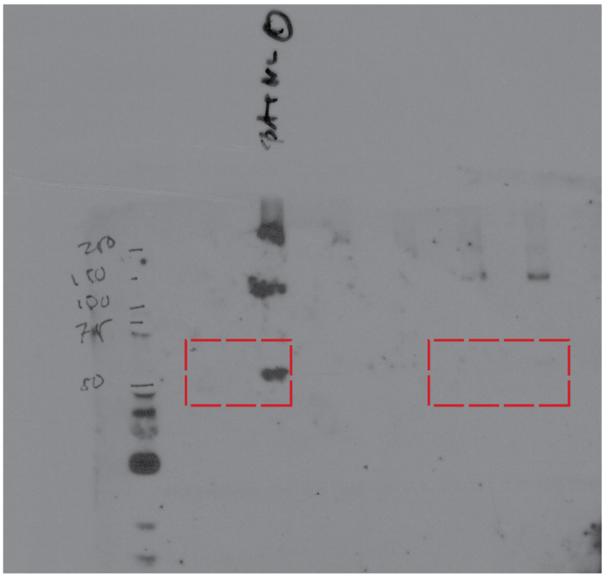

Input: anti-FLAG

anti-tubulin

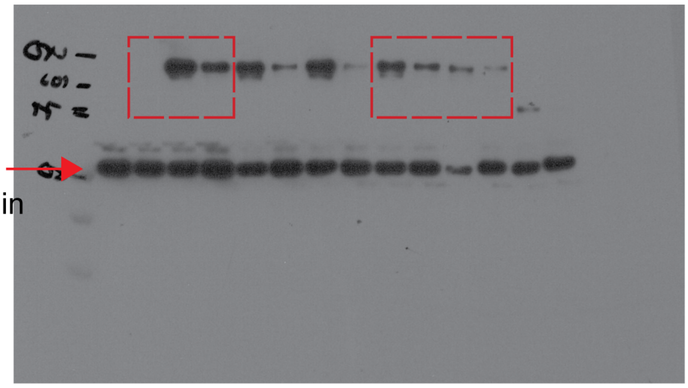

Longer exposure (not shown in the figure)

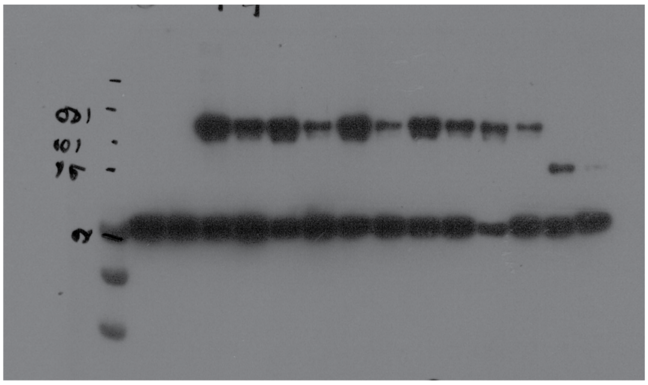

Input: anti-Myc

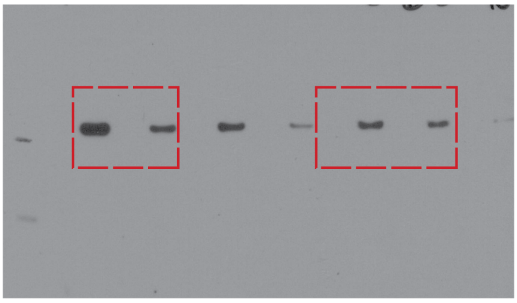

Original blot related to Supplementary Figure 6A

Supplementary Figure 9

Supplementary Table 1: Results of statistical analysis

Figure	Test	Values	Comparison groups
Figure 1C	One-way ANOVA followed by Sidak's multiple comparisons test	ANOVA F(3,12) = 7.209, p = 0.0050	WT vs. C540X, p = 0.0078; WT vs. R660C, p = 0.9252; WT vs. R694C, p = 0.9783; n = 4
Figure 1D	One-way ANOVA followed by Sidak's multiple comparisons test	ANOVA F(3,8) = 21.11, p = 0.0004	Arc vs. WT+Arc, p = 0.0002; Arc vs. R660C+Arc, p = 0.1103; Arc vs. R694C+Arc, p = 0.0510; n = 3
Figure 1F	One-way ANOVA followed by Sidak's multiple comparisons test	ANOVA F(3, 130) = 11.41, p < 0.0001	WT vs. GFP, p = 0.0034; GFP vs. R660C, p = 0.9584; GFP vs. R694C, p = 0.3076; n = 19-49
Figure 2B	One-way ANOVA followed by Sidak's multiple comparisons test	ANOVA F(3,30) = 3.51, p = 0.0271	WT vs. GFP, p = 0.0211; GFP vs. R660C, p = 0.760; GFP vs. R694C, p = 0.9813; n = 8-11
Figure 2E	One-way ANOVA followed by Sidak's multiple comparisons test	ANOVA F(3,126) = 8.83, p < 0.0001	WT vs. GFP, p < 0.001; GFP vs. R660C, p = 0.39; WT vs. R694C, p = 0.907; n = 29-36
Figure 2G	One-way ANOVA followed by Sidak's multiple comparisons test	ANOVA F(3, 126) = 0.18, p = 0.9072	NA. One-way ANOVA is not significant. n = 29-36
Figure 3C	One-way ANOVA followed by Sidak's multiple comparisons test	ANOVA F(3, 53) = 5.13, p = 0.0036	GFP vs. TRIAD3A, p = 0.0405; GFP vs. TRIAD3A+Arc, p = 0.0355; GFP vs. TRIAD3A+Arc-KR, p = 1; n = 12-16;
Figure 3E	One-way ANOVA followed by Sidak's multiple comparisons test	ANOVA F(3, 53) = 2.35, p = 0.083	NA. One-way ANOVA is not significant. n = 12-16
Figure 4C	One-way ANOVA followed by Sidak's multiple	ANOVA F(2, 50) = 6.402, p = 0.0033	Scr vs. TRIAD3A-sh, p = 0.0073; Scr vs. TRIAD3A-sh+TRIAD3A-R, p =

	comparisons test		0.9907; n = 14-27
Figure 4E	One-way ANOVA followed by Sidak's multiple comparisons test	ANOVA F(2, 50) = 2.138, p = 0.1285	NA. One-way ANOVA is not significant. n = 14-27
Figure 4H	One-way ANOVA followed by Sidak's multiple comparisons test	ANOVA F(3, 93) = 7.852, p = 0.0001	Scr vs. TRIAD3A-sh, p = 0.0011; Scr vs. TRIAD3A-sh+R660C, p < 0.0001; Scr vs. TRIAD3A-sh+R694C, p = 0.0020; n = 21-28
Figure 4J	One-way ANOVA followed by Sidak's multiple comparisons test	ANOVA F(3, 92) = 1.649, p =0.1836	NA. One-way ANOVA is not significant. n = 21-28
Figure 5C	Two-way ANOVA followed by Sidak's multiple comparisons test	ANOVA F(1, 20) = 4.676, p = 0.0429	KD vs. Control, Day 1: p = 0.0509; Day 2: p = 0.9210; Day 3: p = 0.4789; Day 4: p = 0.362; n = 11
Figure 5D	Two-way ANOVA followed by Sidak's multiple comparisons test	ANOVA F(1, 20) = 2.062, p = 0.1665	NA. Two-way ANOVA is not significant. n = 11
Figure 5E	Two-way ANOVA followed by Sidak's multiple comparisons test	ANOVA F(1, 20) = 1.498, p = 0.2352	NA. Two-way ANOVA is not significant. n = 11
Figure 5G	Two-way ANOVA with "treatment" and "trials" as factors, with one repeated measure (trials) and animals nested in "treatment", both factors	ANOVA: "treatment" effect: F(1,12)=4.9534, p=0.046;<br>"trial" effect: F(4,258)=7.5699, p=0.000 (not represented, see text for trial effect for each group separately)	"treatment" factor: KD (n=6) vs. Control (n=8);<br>"trials" factor: 4 trials per days over 5 days.

	fixed.		
Figure 5H	One-way ANOVA followed by Bonferroni post hoc multiple comparisons	For Control: ANOVA F(3,28)=20.215, p=0.000 (not represented in the figure) followed by post hoc comparison with quadrant Platform vs. Opp p<0.000, Platform vs. Adj1 p<0.000 and Platform vs. Adj2 p<0.000.<br>For KD: ANOVA F(3,20)=2.465, p=0.092 (not represented in the figure) followed by post hoc comparison with quadrant Platform vs. Opp p=0.137, Platform vs. Adj1 p=0.276 and Platform vs. Adj2 p=1.000.	Number of entries in each of the 4 quadrants of the pool compared with each-other for each of the groups taken separately (KD (n=6) and Control (n=8)).
Figure 5I	One-way ANOVA followed by Bonferroni post hoc multiple comparisons	For Control: ANOVA F(3,28)=6.304, p=0.002 (not represented in the figure) followed by post hoc comparison with quadrant Platform vs. Opp p<0.002, Platform vs. Adj1 p<0.309 and Platform vs. Adj2 p<1.000 (not represented).<br>For KD: ANOVA F(3,20)=4.145, p=0.019 (not represented in the figure) followed by post hoc comparison with quadrant Platform vs Opp p=1.000, Platform vs. Adj1 p=1.000 and Platform vs. Adj2 p=0.352.	Time spent in each of the 4 quadrants of the pool compared with each-other for each of the groups taken separately (KD (n=6) and Control (n=8)).

Figure 6C	One-way ANOVA followed by Sidak's multiple comparisons test	ANOVA F(3,8) = 11.94, p = 0.0025	Arc vs. WT+Arc, p = 0.0040; Arc vs. R660A+Arc, p = 0.9869; Arc vs. R694A+Arc, p = 0.9997; n = 3
Supplementary Figure 1B	One-way ANOVA followed by Sidak's multiple comparisons test	ANOVA F(2,12) = 4.075, p <0.0446	GFP vs. TRIAD3A+Arc, p = 0.0356; GFP vs. TRIAD3A+Arc-KR, p = 0.7645; n = 5
Supplementary Figure 3C	Student's t-test	p = 0.0002	TRIAD3A-sh+Scr vs. TRIAD3A-sh+Arc-sh; n=20
Supplementary Figure 3E	Student's t-test	p = 0.284	TRIAD3A-sh+Scr vs. TRIAD3A-sh+Arc-sh; n = 20
Supplementary Figure 4D	Student's t-test	p = 0.004	TRIAD3A-sh vs. Scr; n = 7-9
Supplementary Figure 5A	Two-way ANOVA followed by Sidak's multiple comparisons test	ANOVA F(1, 20) = 0.1614, p = 0.6921	NA. Two-way ANOVA is not significant. n = 11
Supplementary Figure 5B	Student's t-test	p = 0.6921	KD vs. Control; n = 11
Supplementary Figure 5C	Two-way ANOVA followed by Sidak's multiple comparisons test	ANOVA F(1, 20) = 0.7079, p = 0.4101	NA. Two-way ANOVA is not significant. n = 11
Supplementary Figure 5D	Student's t-test	p = 0.2892	KD vs. Control; n = 11
Supplementary Figure 7C	One-way ANOVA followed by Sidak's multiple comparisons test	ANOVA F(2,26) = 18.19, p <0.0001	WT vs. R660C, p<0.0001; WT vs. R694C, p = 0.0001; n = 9-11
